# Progression to fibrosis and hepatocellular carcinoma in DEN CCl_4_ liver mice, is associated with macrophage and striking regulatory T cells infiltration

**DOI:** 10.3389/fimmu.2025.1601215

**Published:** 2025-07-08

**Authors:** Ananya Ajith, Jonathan Evraerts, Caroline Bouzin, Davide Brusa, Makram Merimi, Mehdi Najar, Françoise Smets, Etienne Sokal, Mustapha Najimi

**Affiliations:** ^1^ Laboratory of Pediatric Hepatology and Cell Therapy, Institute of Experimental and Clinical Research, Université catholique de Louvain (UCLouvain), Brussels, Belgium; ^2^ Institut de Recherche Expérimentale et Clinique (IREC) Imaging Platform (2IP), Institute of Experimental and Clinical Research, Université catholique de Louvain (UCLouvain), Brussels, Belgium; ^3^ CytoFlux-Institut de Recherche Expérimentale et Clinique (IREC) Flow Cytometry and Cell Sorting Platform, Institute of Experimental and Clinical Research, Université catholique de Louvain (UCLouvain), Brussels, Belgium; ^4^ Genetics and Immune Cell Therapy Unit, LBBES Laboratory, Faculty of Sciences, University Mohammed Premier, Oujda, Morocco; ^5^ Osteoarthritis Research Unit, Department of Medicine, University of Montreal Hospital Research Center (CRCHUM), Montreal, QC, Canada; ^6^ Faculty of Medicine, Université Libre de Bruxelles, Brussels, Belgium

**Keywords:** liver, liver fibrosis, hepatocellular carcinoma, immune cells, multiplex immunofluorescence, flow cytometry

## Abstract

**Background and aim:**

Hepatocellular carcinoma (HCC) is a classic inflammation related cancer with most cases arising from chronic liver disease (CLD). This study investigates immune dysregulation that occurs during the progression of CLD to HCC by delineating changes in immune cell composition and distribution within the liver microenvironment.

**Methods:**

Mice were injected with Diethylnitrosamine (DEN) at 4 weeks of age, followed by continuous tri-weekly injections of carbon tetrachloride (CCl_4_) for 6 and 21 weeks to induce liver fibrosis and HCC. Naïve and Phosphate-buffered saline (PBS) corn oil treated mice were used as controls. Immune cell profiling was performed using multiplex immunofluorescence and flow cytometry analyses.

**Results:**

The spatial analysis of immune cell populations in HCC reveals stable leukocytes overall, with notable increases in myeloid cells, particularly infiltrating macrophages (Inf mph). Indeed, Inf mph show a progressive enrichment from control to tumor, reaching a 5-fold and 10-fold increase in the invasive margin (IM) and surrounding non-tumor tissue (NTT) regions, respectively. T lymphocytes, especially CD4+ T cells but not CD8+ T cells, significantly expand, with CD4+ cells increasing up to 10-fold in the IM and NTT regions of HCC livers. Regulatory T cells (Tregs) population exhibits an extraordinary 125-fold and 80-fold surge in the IM and NTT regions, respectively.

**Conclusions:**

The DEN-CCl_4_ induced HCC mouse model replicates key immunosuppressive features of human HCC, notably increased Tregs and macrophages, which provides a robust platform for testing immunotherapies. The prominence of immune cells in the IM region underscores its importance as a critical interface modulating tumor-immune interactions, while the elevated immune presence in the NTT region reflects broader immune dysregulation associated with advanced CLD, and potentially facilitating tumor progression.

## Introduction

Liver cancer is the sixth most prevalent cancer and the third leading cause of cancer-related mortality worldwide (1). Hepatocellular carcinoma (HCC) is a classic inflammation driven cancer (2), with around 80-90% of cases arising in chronic liver disease (CLD) or cirrhosis background (3, 4).

During homeostasis, the liver regulates a tolerogenic condition, to avoid and resolve excessive inflammation and its pathological consequences (5). However, failure to control immune stimulants will transition the liver into a pro-inflammatory state, leading to progressive parenchymal damage and subsequent fibrosis (6, 7). On the contrary, an excessive tolerogenic immune response leads to chronic infection or tumorigenesis (8).

Liver is enriched with innate and adaptive immune cells, that have either protective or detrimental roles during HCC progression (9). Hepatic macrophages include Kupffer cells (KCs) and infiltrating macrophages (Inf mph), and constitute tumor-associated macrophages (TAMs), that promote tumor growth by secreting immunosuppressive mediators able to inhibit the effector functions of CD4 and CD8 T lymphocytes and promote the differentiation of Tregs (10). Previous studies in human have shown that increased infiltration of Tregs has a negative impact on HCC prognosis (11). In addition, TAMs are critical in promoting tumor growth by releasing inflammatory mediators that induce both angiogenesis and metastasis.

Deciphering the dynamic changes in the profile of immune cells during homeostasis and the progression of CLD from liver fibrosis to HCC, remains an area of ongoing investigations that is critical for improving prognostic significance and therapeutic applications.

Diethylnitrosamine (DEN) and carbon tetrachloride (CCl_4_) treated mouse model closely mimics human CLD and HCC progression. DEN activates cytochrome P450 to form alkylating agents, resulting in DNA damage and oxidative stress in the liver (12), while CCl_4_ causes oxidative damage in the liver leading to inflammation and fibrosis (13). Together, DEN serves as the initiator and CCl_4_ acts as the promoting agent for HCC. HCC incidence escalates with the advancing fibrosis stages and increases exponentially during liver cirrhosis (14).

In this study, we used this model to characterize the heterogeneity of the immune microenvironment across healthy, fibrotic, HCC livers.

Although dysregulated immune cell populations have been extensively studied in human and mouse models, our study uniquely tracks the progression of CLD by comparing normal liver, early fibrosis, and HCC stages. By integrating spatial multiplex immunofluorescence with flow cytometry, we delineate immune cell composition and localization, uncovering region-specific immunosuppressive mechanisms that could inform targeted immunotherapies. This comprehensive approach advances our understanding of immune dynamics within tumor microenvironments and highlights the IM as a promising therapeutic target in HCC management.

## Materials and methods

### Animal experiments

Three-week-old C3H/HeOuJ strain male mice (Charles River) were housed under a 12-hour controlled light/dark cycle with unrestricted food and water. The well fare of animals was checked daily.

At 4 weeks of age, mice received a single intraperitoneal injection of 100 mg/kg DEN diluted in phosphate-buffered saline (PBS) to induce oncogenic mutations. Beginning at 8 weeks of age, mice were administered 0.5 ml/kg CCl_4_ diluted in corn oil, three times a week. Mice treated with CCl_4_ for 6 weeks (n=14) were used to promote liver fibrosis, whereas those treated for 21 weeks (n=7) developed HCC on the background of advanced CLD. Control groups included naïve mice (n=4) for flow cytometry and PBS-corn oil-treated mice (n=5) for multiplex immunofluorescence (IF). These controls were chosen to ensure that observed outcomes are attributed to the chemical treatments rather that vehicle components.

Animal welfare was monitored daily, and all experiments followed Royal Decree guidelines on experimental animal protection and approved by the institution animal ethic review board (2022/UCL/MD/59).

### Sirius Red and Hematoxylin-Eosin Staining

Formalin-fixed, paraffin-embedded (FFPE) liver samples were sectioned into 5 μm slices for Sirius Red and Hematoxylin-Eosin (H&E) staining. Slides were immersed in toluene (three cycles, 5 minutes each), blot-dried on absorbent paper, then immersed in isopropanol (three cycles, 5 minutes each). After rinsing with running and distilled water, Sirius Red staining was performed by immersing slides in 2% phosphomolybdic acid for 2 minutes, followed by staining with Direct Red 80 for 2 hours in the dark. Slides were then treated with 0.01N HCl for 2 minutes, rinsed with distilled water and methanol, immersed in xylene (three cycles, 5 minutes each). Slides were mounted with Dako coverslips and digitalization using a Pannoramic ScanII slide scanner.

For H&E staining, slides followed the same initial steps with toluene, isopropanol, and water rinses. Slides were then stained with Mayer’s Hematoxylin for 10 minutes, rinsed in running and distilled water (5 minutes each), and stained with 0.5% aqueous Eosin for 4 minutes. After quick water rinses, slides were air-dried for 15 minutes and mounted using Sakura Tissue-Tek Film and digitalization using a Pannoramic ScanII slide scanner.

### Multiplex immunofluorescence

For histological and immunofluorescence analysis, FFPE liver samples from control, fibrotic (6 weeks), and HCC (21 weeks) groups were sectioned at 5 µm thickness and mounted on positively charged slides. After deparaffinization, and rehydration using toluene and isopropanol, liver sections were incubated with 3% hydrogen peroxide solution for 20 minutes at room temperature to block endogenous peroxide activity. Heat-induced epitope retrieval (HIER) was performed using citrate (pH 5.7) or Ethylenediaminetetraacetic acid (EDTA) (pH9) solutions in microwave followed by blockade of non-specific antigen binding sites (Tris buffered saline containing 0.1% Tween-20 (TBST) and 5% Bovine Serum Albumin (BSA)). Primary antibodies were incubated for 1 hour at room temperature in TBST containing 1% BSA and detected by corresponding horseradish peroxidase (HRP)-conjugated polymer secondary antibodies (Envision, Agilent #K4001 or K4003) for 40 min at room temperature. HRP was then visualized by tyramide signal amplification (TSA) using CF-conjugated tyramides. After a new HIER incubation step (which detaches antibodies), the same protocol was applied with other primary antibodies and different CF or AF-conjugated tyramides. Antigens and target cells of this study are indicated in **Supplementary Table S1**. Immune cell types analyzed in 4 multiplex panels are indicated in **Supplementary Table S2**. After a washing step in TBST, nuclei were finally stained with Hoechst 33342 (Sigma #1453) diluted in distilled water and mounted with Dako fluorescence mounting medium (Agilent #S3023). Experiments were conducted in IREC Imaging Platform 2IP (RRID: SCR_023378)

### Image acquisition and computer-assisted quantitative evaluation

For multiplex immunofluorescence, whole slide scans of the stained tissue sections were captured using AxioScan.Z1 scanner (Zeiss, Oberkochen, Germany) with X20/0.8 Plan-Apochromat objective using Zen-blue acquisition software, for the identification of up to 7 stains (6 markers and nuclear dye). Image analysis was performed using Qupath 0.5.1 software. Additional information about annotating the region of interest and cell detection is included in the **Supplementary Document** (**Supplementary Figure S1**).

### Hepatic non-parenchymal cell isolation

Mice were anesthetized using xylazine (100mg/kg) and ketamine (10mg/kg). Liver enzymatic digestion began with perfusion through the upper vena cava via the right atrium using HBSS buffer (without Ca²^+^, Mg²^+^), followed by a second perfusion with collagenase P (1 mg/ml in HBSS with Ca²^+^, Mg²^+^) after clamping the inferior vena cava infrarenal at 37°C. Undigested tumor tissue of HCC liver was subjected to an additional step of mechanical digestion. Single cell suspension of the digested liver was passed through a 100μm cell strainer. Cell suspension was centrifuged 3 times at 50g (4°C, 3 minutes) to separate hepatocytes (pellet) from the supernatant containing non-parenchymal cells (NPCs). NPCs were further centrifuged 3 times, at 300g (4°C, 10 min) and treated with mouse RBC lysis buffer (Thermofisher) to eliminate contaminating red blood cells. Cell counts and viability (>85%) were assessed via Trypan blue exclusion.

### Flow cytometry analysis

At the 6-week fibrosis stage, one million NPCs were used for flow cytometry. After PBS washing, cells were incubated with 10 μl/ml FcR blocking reagent (Miltenyi) at 4°C for 10 minutes, followed by staining with fluorochrome-conjugated antibodies (CD45, CD11b, F4/80, Ly6G, Ly6C, CD3, CD4, CD8; **Supplementary Table S2**) for 10 minutes at room temperature. To analyze Tregs, intercellular FoxP3 staining was performed after fixing the cells in fixation/permeabilization buffer (FoxP3 staining buffer set, Miltenyi). For unfixed cells, DAPI was used as the Viability dye. The immune cell population analysis was performed using Novocyte Quanteon system and data analysis by FlowJo software. Experiments were conducted in CytoFlux-IREC Flow Cytometry and Cell Sorting Platform (RRID: SCR_023664).

### Statistical analysis

Data are represented as mean ± Standard Deviation. One-way analysis of variance followed by *post-hoc* Tukey test with two-tailed distribution (p<0.05, *p<0.01, **p<0.001) was performed for 3 or more groups. Descriptive fold-changes were calculated by dividing group means relative to control values as commonly applied in spatial immune profiling studies (11). Correlation matrices were performed using spearman correlation coefficients. All statistical analyses were performed using Graph-pad Prism software (version 10.2).

## Results

### Chronic liver disease progression and HCC tumor development in DEN and CCl_4_ treated mice

**Figure 1:**
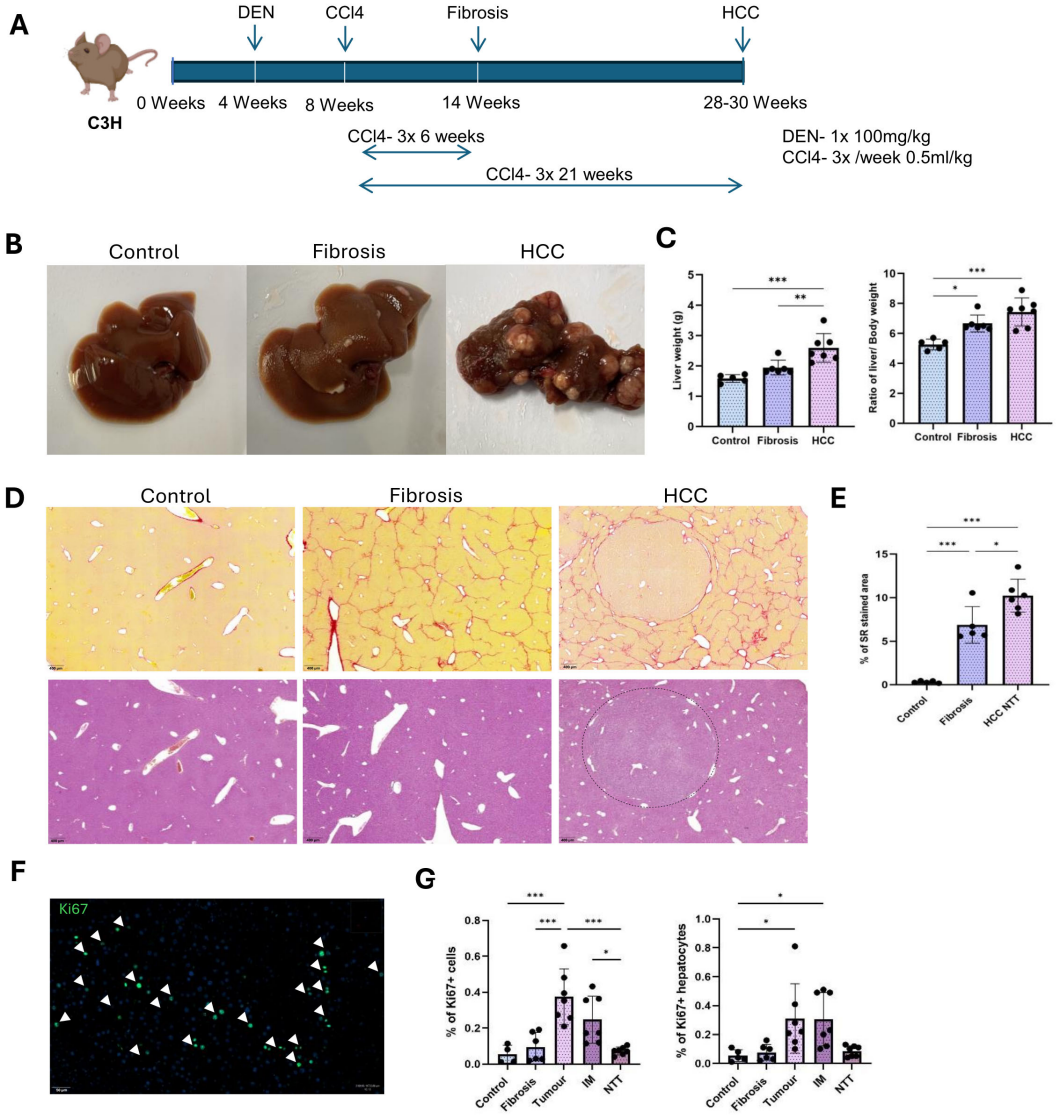
DEN and CCl4 induced liver fibrosis and HCC in mice. **(A)** Single injection of 100mg/kg DEN at 4 weeks of age, followed by CCl4 injections of 0.5ml/kg, 3 times a week for 6 weeks to induce liver fibrosis and for 21 weeks to induce the development of HCC tumors in the background of CLD. **(B)** Representative macroscopic liver images of control, fibrosis and HCC livers. **(C)** Change in the liver weight and liver to body weight ratios between control (n=5), fibrosis (n=6), HCC (n=7). **(D)** Representative images of Sirius red and Hematoxylin-eosin staining on control, fibrosis and HCC livers. Collagen encapsulated tumor in HCC liver by the Sirius red staining. **(E)** Percentage of Sirius red-stained area in control (n=5), fibrosis (n=5) and HCC-NTT (n=6). **(F)** Ki67^+^ proliferating cells in tumor. **(G)** Percentage of proliferating cells to total Hoechst detection and percentage of proliferating hepatocytes to total hepatocyte population in control (n=4), fibrosis (n=4), tumor, IM and RT regions of HCC livers (n=4). Error bars represent mean ± SD. One-way ANOVA followed by Tukey’s multiple comparison test was performed. *p<0.05, **p<0.01, ***p<0.001.

**Figure 1A**, shows the detailed DEN and CCl4 injections for inducing liver fibrosis and HCC in C3H/HeOuJ mice strain. All treated mice exhibited significant liver morphological changes and developed HCC within 30 weeks of age (**Figure 1B**). Relative weights increased as follows: 1.6 ± 0.12 g (healthy), 1.95 ± 0.22 g (+22%, fibrosis), and 2.47 ± 0.36 g (+54.4%, HCC) alongside higher liver to body weight ratio: (5.2 ± 0.32% in healthy controls, 6.67 ± 0.5% in fibrotic and 7.24 ± 0.84% in HCC groups) (**Figure 1C**).

Histological assessment by Sirius red staining revealed well extended collagen networks between central veins (**Figure 1D**). Quantification by Qupath analysis showed significantly increased collagen deposition (0.32 ± 0.1% in healthy controls, 6.8 ± 1.9% in fibrotic and 10.2 ± 1.7% in non-tumor region of HCC group) (**Figure 1E**). In the HCC group, tumor nodules were surrounded by collagen and the increased collagen deposition in the non-tumor tissues (NTT) indicated tumor development in the setting of advanced CLD (**Figure 1D**). By Hematoxylin and eosin staining, tumor cells were differentiated from the remaining hepatocytes based on their smaller cell size (**Figure 1D**). Cell proliferation marker Ki67, was used to analyze the percentage of proliferating cells (15) in each experimental condition (**Figure 1F**). Significantly increased rate of proliferating cells was observed within the tumor (0.4 ± 0.14% of total Hoechst detection) and invasive margin (IM) regions (0.25 ± 0.12% of total Hoechst detection) compared to healthy (0.05 ± 0.04% of total Hoechst detection) and fibrotic (0.1 ± 0.07% of total Hoechst detection) livers as well as the NTT regions (0.07 ± 0.02% of total Hoechst detection) of the HCC group (**Figure 1G**). Likewise, the rate of proliferating hepatocytes, which were identified as Ki67 and hepatocyte nuclear factor 4 expressing cells, was also observed to be highest in the tumor and IM regions with an average of 0.3 ± 0.2% and 0.3 ± 0.17 to total hepatocyte population (**Figure 1G**).

### Heterogenous tissular infiltration of leukocytes and myeloid cells in fibrotic and HCC livers

**Figure 2:**
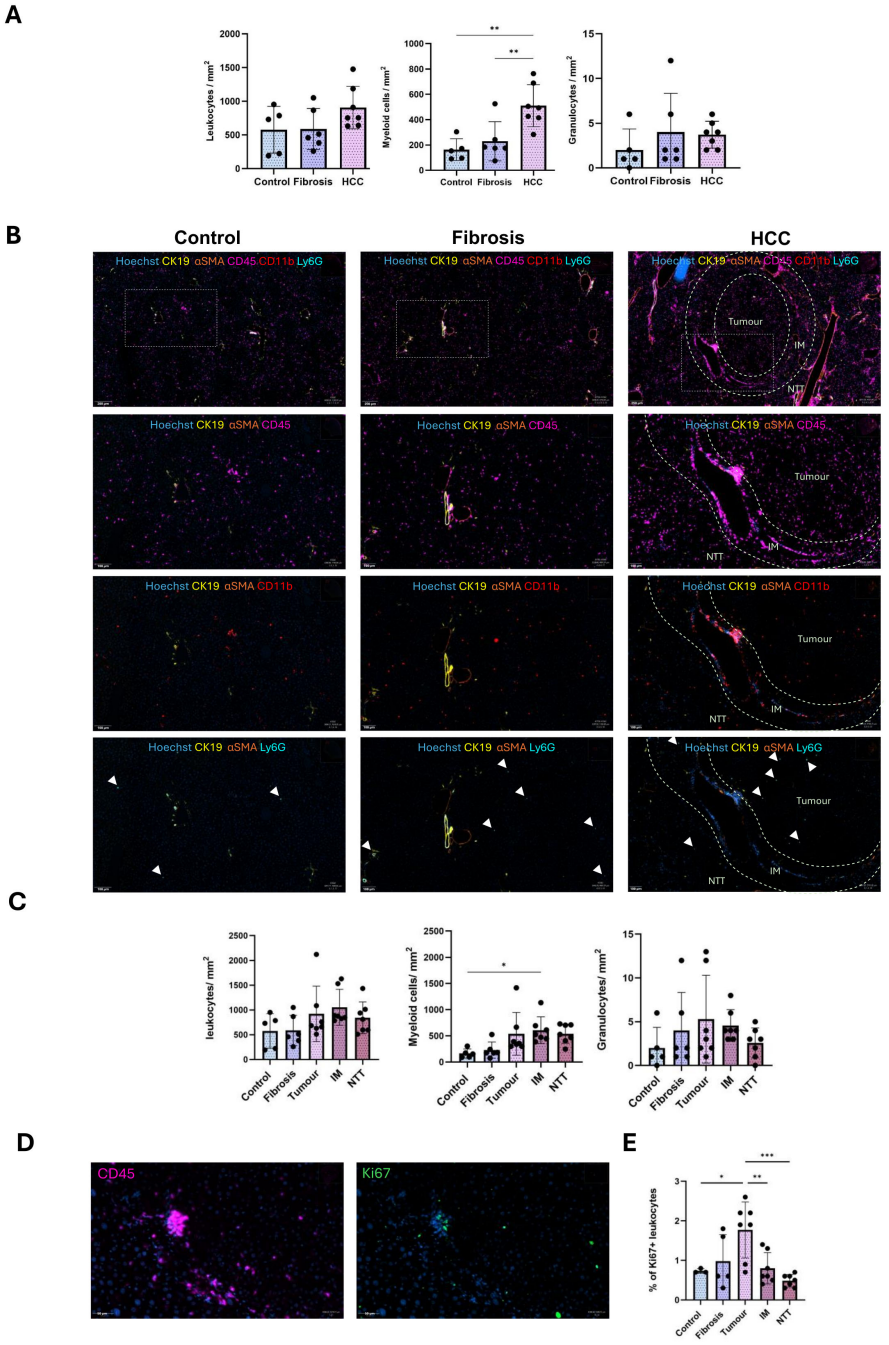
Altered spatial localization of immune cells during CLD progression from fibrosis to HCC. **(A)** Quantification of the number of leukocytes, myeloid cells, granulocytes per mm^2^ tissue in control, fibrosis and HCC livers (combined all 3 regions). **(B)** Representative staining images of CD45 leukocytes, CD11b myeloid cells and Ly6G granulocytes in control, fibrosis and HCC (tumor, IM and RT regions) groups. **(C)** Quantification of the number of leukocytes, myeloid cells and granulocytes per mm^2^ tissue in control (n=5), fibrosis (n=6), tumor, IM and NTT of the HCC (n=7) group. **(D)** Representative immunostaining image of Ki67^+^ proliferating CD45 leukocytes in the tumor region of HCC liver. **(E)** Percentage of proliferating CD45 leukocytes to total leukocyte population. Error bars represent mean ± SD. One-way ANOVA followed by Tukey’s multiple comparison test for each individual immune cell population was performed. *p<0.05, **p<0.01, ***p<0.001.

To characterize the spatial distribution of these immune cell populations, we performed multiplex IF staining on FFPE tissue sections in corn-oil treated control, fibrotic and HCC livers. Quantification of leukocytes, myeloid cells and granulocytes per mm^2^ of tissue in controls, fibrotic and HCC livers (combined all 3 regions), revealed a non-significant increase of leukocytes and granulocytes, but a significant increase of myeloid cells (**Figure 2A**).

Analysis of the HCC microenvironment HCC (tumor, IM, and NTT regions), revealed that the overall leukocyte (CD45) distribution remains relatively stable across healthy, fibrotic and HCC livers (**Figures 2B, C**), while proliferating leukocyte populations were significantly upregulated only in the tumor region (**Figures 2D, E**). Conversely, increased hepatic myeloid cell presence (CD11b) was observed only in the IM region with no changes noted for low-level granulocytes (Ly6G) (**Figures 2B, C**).

### Shift in the hepatic macrophage population from resident Kupffer cells to infiltrating macrophages in fibrotic and HCC livers

KCs and Inf mphs were identified based on the C-Type Lectin Domain Family 4-member F (CLEC4F) and Ionized Calcium Binding Adaptor molecule 1 (IBA1) cell surface expression by multiplex analysis. IBA1 is expressed in all monocytes/macrophages while CLEC4F is specifically expressed only on KCs. All hepatic macrophages were IBA1^+^ cells, KCs were IBA1^+^ CLEC4F^+^ and Infiltrating macrophages (Inf mphs) were identified as IBA1^+^ CLEC4F^-^.

Cumulated quantification of hepatic macrophages, KCs, and Inf mph per mm^2^ tissue in control, fibrotic and HCC liver (combined all 3 regions), revealed an increase which became significant in HCC liver as compared to controls and fibrotic livers.

**Figure 3:**
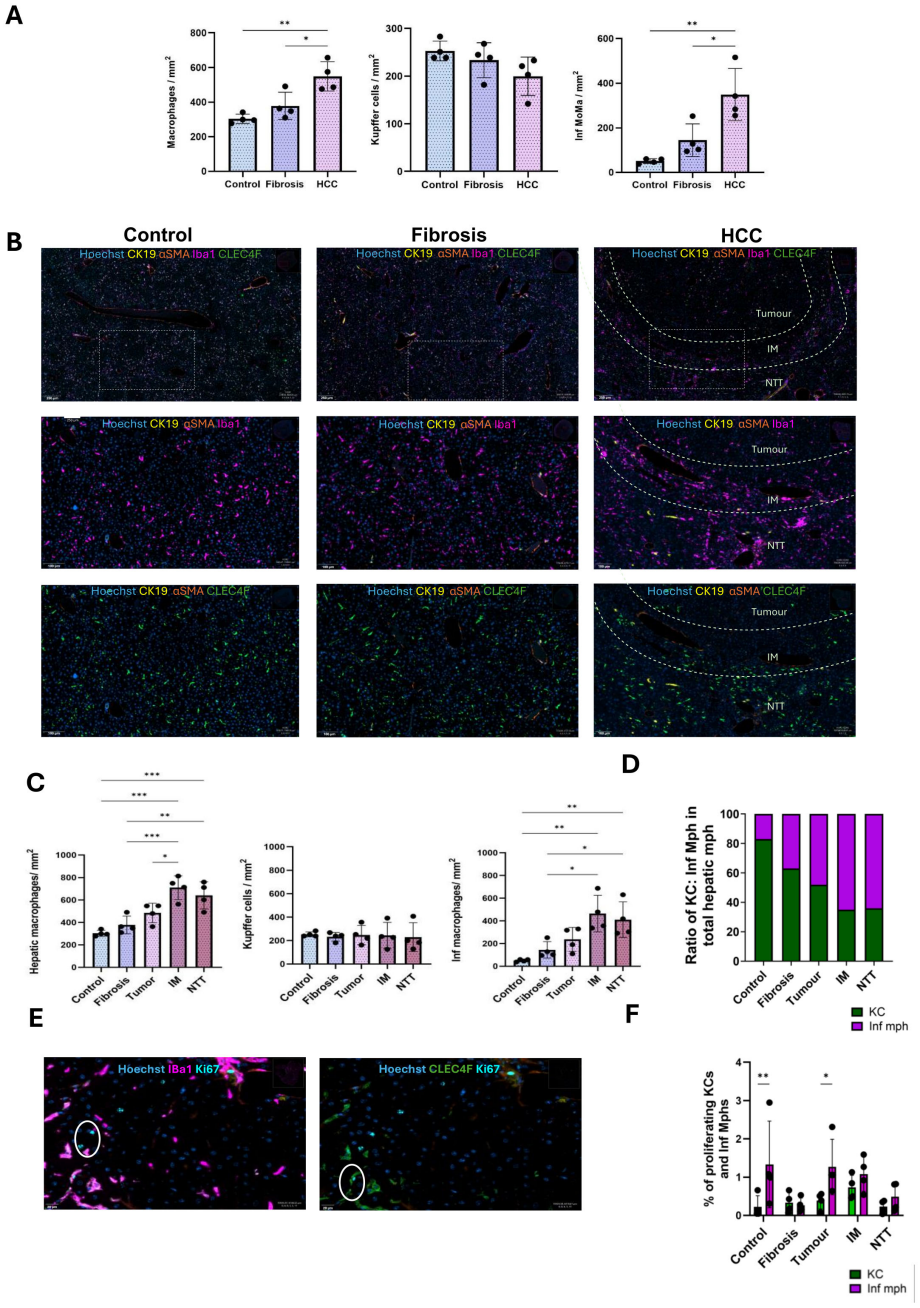
Increased numbers of infiltrating macrophages during CLD progression from fibrosis to HCC. **(A)** Quantification of the number of macrophages, Kupffer cells, Inf macrophages per mm^2^ tissue in control, fibrosis and HCC livers (combined all 3 regions). **(B)** Representative immunostaining images of IBA1^+^ macrophages and CLEC4F^+^ KCs in control, fibrosis and HCC (tumor, IM and RT regions) groups. **(C)** Quantification of the number of hepatic macrophages, KCs and Inf mph per mm^2^ tissue in control, fibrosis groups and tumor, IM and RT of the HCC group (n=4). **(D)** Ratio of KCs and Inf Mphs in total hepatic macrophage populations in all 5 regions. **(E)** Representative immunostaining images of Ki67^+^ proliferating KCs and Inf Mphs. **(F)** Percentages of proliferating KCs and Inf Mphs to total KC and Inf mphs populations, respectively. Error bars represent mean ± SD. One-way ANOVA followed by Tukey’s multiple comparison test was performed. *p<0.05, **p<0.01, ***p<0.001.

There was no change in the KCs population across the three conditions. However, an increase in the Inf mphs was evident especially in HCC liver (**Figures 3A, B**). In comparison to the control and fibrotic livers, these cells were more abundant in the IM and NTT regions of HCC liver (**Figures 3B–D**). Only a small proportion of KCs and Inf mphs were in proliferative condition. (**Figures 3E, F**). In healthy liver, Inf mphs were comparatively more proliferative than KCs. The same was the case in tumor and IM regions of the HCC liver, but in fibrosis and advanced fibrosis environment of NTT region, both cells had a similar rate of proliferation (**Figures 3E, F**).

### Increased infiltration of T lymphocytes in HCC liver

**Figure 4:**
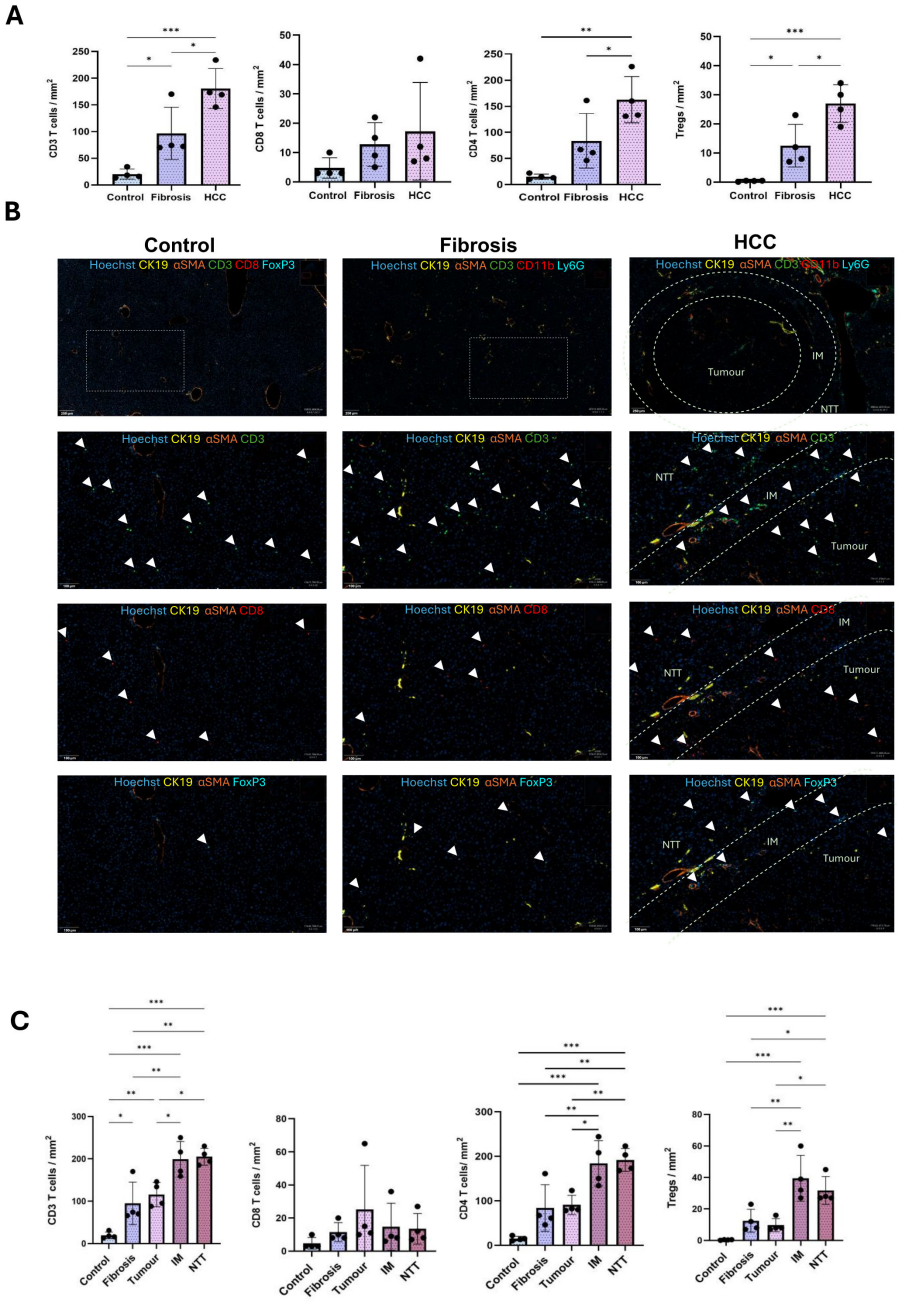
Increased infiltration of T lymphocytes during CLD progression from fibrosis to HCC. **(A)** Quantification of the number of CD3 T, CD8 T, CD4 T cells and Tregs per mm^2^ tissue in control, fibrosis and HCC livers (combined all 3 regions). **(B)** Representative immunostaining images of T lymphocytes like, CD3 T, CD8 T, CD4 T cells and Tregs in control, fibrosis and HCC (tumor, IM and RT regions) groups. **(C)** Quantification of number of CD3 T, CD8 T, CD4 T cells and Tregs per mm^2^ tissue in control, fibrosis groups and tumor, IM and NTT of HCC group (n=4). Error bars represent mean ± SD. One-way ANOVA followed by Tukey’s multiple comparison test was performed. *p<0.05, **p<0.01, ***p<0.001.

Global quantification of CD3+ T cells, CD8+ T cells, CD4+ T cells, and Tregs per mm^2^ tissue in control, fibrotic and HCC livers (combined all 3 regions), revealed a significant increase in the CD3+ T lymphocytes as the disease progressed from fibrosis to HCC. Although no significant change was observed in the CD8+ T cells, CD4+ T cells increased across the three conditions and the increase was even more prominent for Tregs as the disease progressed from fibrosis to HCC (**Figure 4A**).

The increase in T cells was more prominent in the IM and NTT regions compared to the tumor tissue in HCC liver (**Figures 4B, C**).

CD3+ T cells were significantly more abundant in the IM and NTT regions of HCC liver, with a higher proportion of CD4+ T cells than CD8+ T cells. The CD4+ T cell increase was not significant in the fibrotic livers but significant in the IM and NTT regions of HCC livers. Tregs, which constitute a minor cell population in naïve liver, had a non-significant global increase in fibrotic livers, and a significant increase in HCC livers, with the most abundant concentration in the IM and NTT regions, compared to tumor tissue (**Figures 4B, C**).

### Hepatic immune landscape and interaction among different immune cell types

**Figure 5:**
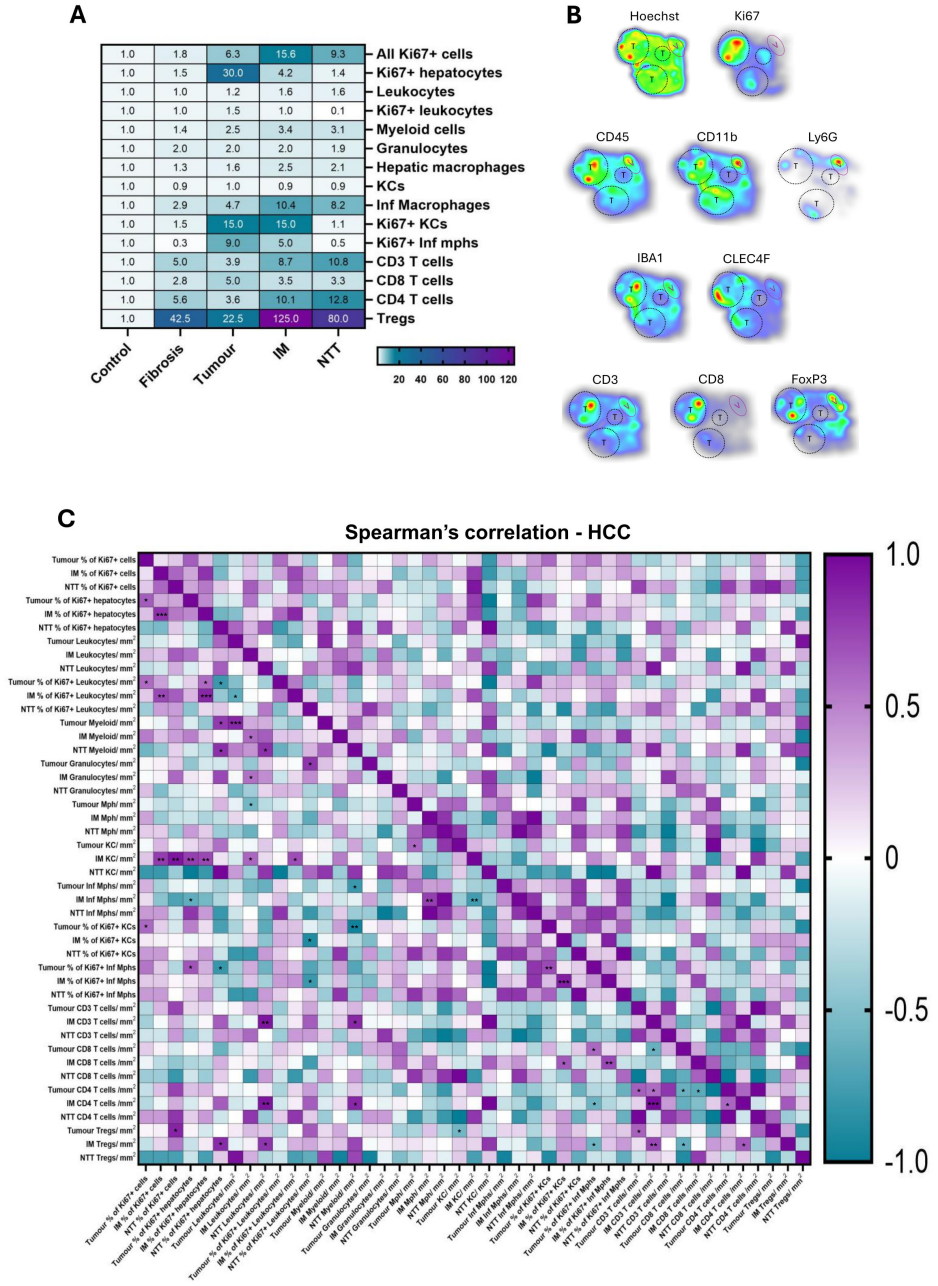
Infiltration and interaction among different immune cell types differs in healthy, fibrosis and HCC livers. **(A)** Heatmap showing the fold change (against the control group) of immune cell populations in the studied groups. **(B)** The densities of various immune cells analyzed through multiplex IF are presented as a heatmap in HCC liver tissue. Red hues indicate high cell density while blue ones indicate low cell density. Tumor areas are depicted by black dash circles and tumor regions are depicted by purple oval. **(C)** Spearman’s correlation matrix and respective p-values of the different parameters stained on HCC liver (tumor, IM, NTT). *p<0.05, **p<0.01, ***p<0.001.

The average values of immune cell variables in fibrotic and HCC groups (tumor, IM, and NTT regions) were compared to the control group to calculate the fold change (**Figure 5A**). A 30-fold increase in proliferating hepatocytes was detected in HCC tumors. No significant fold change in the hepatic leukocytes, myeloid cells and granulocytes were observed between the study groups. However, macrophage populations exhibited significant variation. Kupffer cells (KC) populations remained stable, while infiltrating macrophages (Inf mph) increased markedly, rising approximately 3-fold from control to fibrotic livers, 5-fold in tumor regions and 10-fold in the IM and NTT regions of HCC livers. CD3+ T lymphocyte populations showed a progressive increase, particularly CD4+ T cells, which exhibited a 5-fold increase in fibrosis and a 10-fold increase in the IM and NTT regions of HCC livers compared to controls. Among the different immune cells analyzed, Tregs demonstrated the most substantial rise, increasing 125-fold in the IM region and 80-fold in the NTT regions of HCC livers. These findings indicate significant immune cell redistribution and activation during CLD progression, with infiltrating macrophages and Tregs predominantly accumulating in the IM region (**Figure 5A**).

The heatmap in **Figure 5B** illustrates the heterogeneous distribution of immune cells across HCC tumors, highlighting variations in cellular proliferation and immune infiltration. Immune cell density was influenced by tumor size, with larger tumors exhibiting higher concentration of immune cells. The correlation between different immune variables was analyzed by spearman’s correlation in tumor, IM, and NTT regions of HCC livers. The heatmap presented here (**Figure 5C**) have correlations ranging from -1, a perfect negative correlation, to +1, a perfect positive correlation, and their corresponding p-value significance. **Supplementary Table S4** reports the complex correlations between different immune cell populations of HCC livers in the tumor region between proliferating hepatocytes and proliferating Inf mph (r= 0.67, p= 0.012), tumor CD8+ T cells and proliferating Inf mph (r= 0.60, p= 0.032), and KCs and Tregs (r= -0.56, p= 0.048) as well as in the IM region, between KCs and proliferating hepatocytes (r= 0.72, p= 0.0096), CD8+ T cells and proliferating Inf mph (r= 0.73, p= 0.009), tumor CD8+ T cells and IM Treg (r= -0.63, p= 0.023). More complex correlations in the HCC liver are depicted in **Supplementary Table S4**.

### Differential dysregulation of immune cell populations in fibrotic livers

To validate multiplex IF results, we performed flow cytometry to characterize various hepatic immune cell populations in NPC suspensions isolated from naïve and fibrotic livers. However, significant heterogeneity in tumor nodule size made it impractical to dissect individual nodules, as distinguishing true tumor nodules from fibrotic and cirrhotic tissue was challenging. Additionally, tumor number and size varied among mice. Moreover, an additional mechanical dissociation step, alongside collagenase perfusion, was required to process larger tumors. For these reasons, digesting entire livers along with tumor nodules proved inefficient, and therefore, those results are not presented here. Therefore, flow cytometry analysis was limited to the 6-week time point, when liver tissue was more uniform and technically suitable for reliable immune profiling

**Figure 6:**
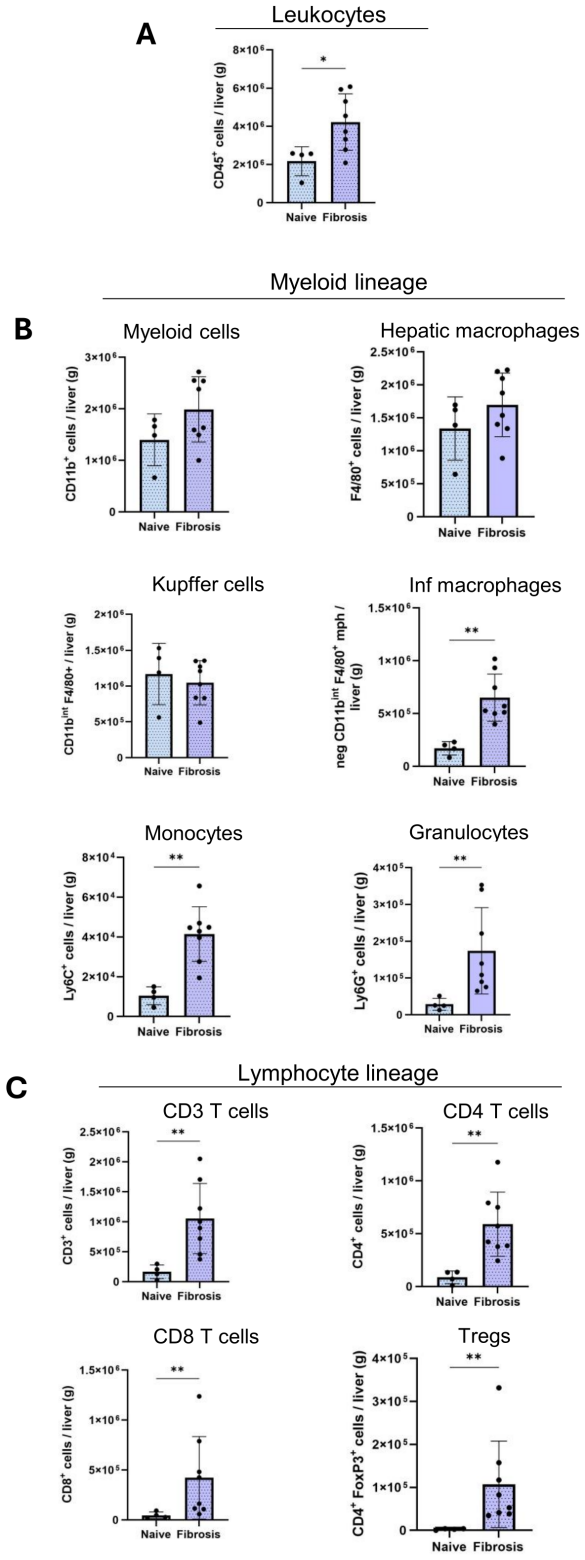
Altered infiltration of immune cells in fibrosis livers. Quantification of the absolute cell numbers of several immune cell populations per g of liver by flow cytometry. **(A)** Leukocytes, **(B)**, Myeloid cell lineage, **(C)**, Lymphoid cell lineages in naïve (n=4), fibrosis (n=8) groups. Error bars represent mean ± SD. One-way ANOVA followed by Tukey’s multiple comparison test was performed. *p<0.05, **p<0.01, ***p<0.001.

Consistent with our multiplex IF results, flow cytometry data (**Figure 6**) revealed a significant increase in intrahepatic leukocyte populations (CD45^+^) in the fibrotic livers (4×10^6^ ± 1.0×10^6^ cells/g) compared to naïve (2×10^6^ ± 0.07×10^6^ cells/g) livers (**Figure 6A**). Myeloid cells (CD11b^+^) and hepatic macrophages (F4/80^+^) did not show significant dysregulation during fibrosis (**Figure 6B**). Macrophage populations largely consist of resident KCs (F4/80^high^ CD11b^int^) and Inf mphs (F4/80^low/high^ CD11b^high^) (**Figure 6B**). KC numbers had a non-significant decrease in fibrotic livers (1×10^6^ ± 0.3×10^6^ cells/g) compared naive (1×10^6^ ± 0.4×10^6^ cells/g) livers (**Figure 6B**). Meanwhile, Inf mphs increased markedly in fibrotic livers (6.5×10^5^ ± 0.2×10^5^ cells/g) compared to naive liver (1.7×10^5^ ± 0.6×10^5^ cells/g) (**Figure 6B**). Similarly, hepatic monocyte numbers (CD11b^+^ Ly6C^+^) increased significantly in fibrotic (4.2×10^4^ ± 1.6×10^4^ cells/g) compared to naïve livers (2×10^4^ ± 2.5×10^4^ cells/g), and granulocytes (CD11b^+^ Ly6G^+^) also followed a similar trend during fibrotic (2×10^5^ ± 1.2×10^5^ cells/g) than naive livers (0.4×10^5^ ± 0.3×10^5^ cells/g) (**Figure 6B**).

T lymphocyte cell populations (CD3+) increased significantly during fibrosis (1.2×10^6^ ± 0.5×10^6^ cells/g) and CD4+ T cells were confirmed more abundant than CD8+ T cells in liver. CD4+ T cells increased from (0.8×10^5^ ± 0.5×10^5^ cells/g) in naïve liver to (6×10^5^ ± 2.6×10^5^ cells/g) in fibrotic liver (**Figure 6C**). Similarly, we observed the same significant increase during fibrosis in CD8+ T cells (0.4×10^5^ ± 0.2×10^5^ cells/g, 4×10^5^ ± 4×10^5^ cells/g) and Tregs (0.02×10^5^ ± 0.01×10^5^ cells/g, 1×10^5^ ± 0.9×10^5^ cells/g) populations (**Figure 6C**).

## Discussion

Our data using the DEN-CCl_4_ treated mouse model provide an original longitudinal dynamic observation of the changes in the liver immune microenvironment- from healthy livers to fibrosis and HCC- mimicking the natural history of human CLD. The gene expression pattern in the DEN mouse model closely resembles that of human HCC with poor survival outcomes (16). Recent proteomic analyses have further confirmed that this HCC model, is linked to Human HCC showcasing proliferation, aggressiveness and heightened immune infiltration (12). In this study, we aimed to characterize the immune microenvironmental changes during CLD progression from fibrosis towards HCC and relate them to known immune subclasses of human HCC, highlighting the relevance of this model for translational research. Combining DEN to an additional fibrogenesis stimulus like CCl_4_ induces a quicker tumorigenesis with a more prominent inflammatory response (17).

The immune cell dysregulation in fibrotic livers was characterized by increased populations of hepatic leukocytes, myeloid cells, macrophages, and Tregs (18, 19) and we show that the same trend continued as the disease progressed to HCC. Multiplex IF imaging illustrated best the transition of the immune landscape showing spatial localization of immune cells during liver disease progression. We also subdivided the HCC liver parenchyma into three regions: tumor, IM, and NTT. The IM, where immune cells interact with the tumor, is crucial for immune surveillance and tumor progression (20, 21) while the NTT region reflects advanced fibrosis exempt of tumor.

The dynamic interactions between the tumor cells, immune cells and immunomodulators within the tumor microenvironment can either suppress or enhance the immune response (22), and HCC progression is favored by the immunosuppressive niche created by the fibrogenic microenvironment of a cirrhotic liver (23). Tumor associated immune cells are broadly classified into pro-tumor and anti-tumor immune cells (24). An increase in Tregs, and M2 macrophages (anti-inflammatory), is known to be associated with HCC tumor progression, whereas an abundance of CD8+ T cells and M1 (pro-inflammatory) macrophages are associated with good prognosis of patient survival (25).

KCs and Inf mphs, play an important role in chronic liver inflammation and tumor microenvironment, and increase during the progression from healthy to fibrotic and to HCC livers (26). Previous studies have shown that TAMs, comprising both intra-tumoral KCs and Inf mph, are abundantly present in the HCC microenvironment and act as positive regulators of tumor progression (27–29). TAMs have a dual role in both supporting and restricting tumor growth and metastasis (30, 31). While we did not perform direct functional studies, we employed Spearman’s correlation analyses to understand the functions, based on immune cell interactions and hepatocyte proliferation. In the tumor region, both proliferating KCs and Inf mphs positively correlated with proliferating hepatocytes and with each other, suggesting a tumor-promoting role. However, Inf mphs also showed a positive correlation with CD8+ T cells, while KCs negatively correlated with Tregs, indicating possible anti-tumor phenotype. In the IM, KCs were positively correlated with both proliferating hepatocytes and CD8+ T cells, again suggesting their dual role in tumor growth and development. Inf mphs were negatively correlated to the proliferating hepatocytes implying an anti-tumor effect. Meanwhile a negative correlation between the Inf mph and KCs here could indicate a competitive or divergent role of these macrophage subsets in this region. Collectively our data suggests that TAMs exhibit dual functional roles in tumor and IM regions possibly promoting and restricting tumor growth and cell proliferation. Future studies involving M1/M2 marker expression or functional assays (e.g., cytokine profiling) would be valuable to confirm and expand these observations.

In the NTT, enrichment of inf mphs likely reflects an ongoing immune dysregulation linked to persistent inflammation and advanced fibrosis. Altered fibrotic stroma could change the macrophage behavior (32). During fibrosis, hepatic stellate cells secrete chemokines such as CCL2 that promote monocyte/macrophage recruitment (33), suggesting a fibrosis-driven chemotactic axis for Inf mph localization. Similarly, in our model due to CCl_4_ treatment centrilobular hepatocyte death and extracellular matrix deposition (34), longitudinal increase of Inf mphs specifically in the central vein (CV) zones of fibrosis and NTT regions of HCC livers was observed (**Supplementary Figure S2D**). Moreover, monocytes/macrophages in the peritumoral stroma (NTT) have been reported to exhibit an activated phenotype, that suppress tumor-specific T cell responses and to be associated with poor prognosis in HCC (35). The tumor core typically evolves into an immunosuppressive, hypoxic microenvironment that impairs immune cell infiltration and function. Hypoxia can reprogram macrophage metabolism, pushing them toward an M2-like, tumor-promoting phenotype due to IL-4 and IL-10 secretions or impairing their survival (36). Enrichment of Inf mph in the IM than in tumor region further reflects its role as a dynamic interface where recruited macrophages interact with both tumor and fibrotic stroma. Macrophages in the IM may play a unique role by potentially balancing immune surveillance and supporting tumor progression. Therefore, the spatial distribution of Inf mphs in our model could possibly be driven by fibrosis-associated chemokine gradients and structural or metabolic constraints within the tumor microenvironment. Future studies incorporating chemokine profiling and hypoxia markers will help validating these spatial observations.

Tumor infiltration of cytotoxic CD8+ T cells is a favorable prognostic marker in various cancers (37), as they induce cancer cytotoxicity by producing interferons and tumor necrosis factors (24). Hence, the reduced infiltration, dysfunction and inhibition of CD8+ T cells in the HCC microenvironment may result in tumor immune escape (38). In our model, the lack of change in infiltrating CD8+ T cells as the disease progressed from fibrosis to HCC, especially in the intratumoral region, is indicative of an immunosuppressive tumor microenvironment.

Higher infiltration of CD4+ T cells, crucial for anti-tumor immunity (39), was observed in fibrosis, and in the IM and NTT regions of HCC liver. CD4+ T cells are mainly composed of CD4+ T helper and Treg cells (40). In our model, the most substantial change was observed in the Treg infiltration, which increased markedly from healthy to fibrotic to HCC livers. In HCC livers, Tregs were predominantly localized in the IM region, followed by the NTT) region, reflecting their significant presence in areas of tumor invasion and advanced fibrosis. The highest Treg infiltration in the IM region of HCC livers, likely reflects its role as a transitional zone between tumor and fibrotic tissue. This area may act as an immunological interface where immune surveillance is subverted to promote tumor growth. The overall increase in Tregs within the tumor microenvironment is driven by TGFβ-induced polarization of naïve CD4+ T cells (41). In solid tumor, chemokines like CCL22/CCR4, CCL5/CCR5, CCL28/CCR10, and CXCL12/CXCR4 facilitate the recruitment and proliferation of thymus-derived Tregs (tTregs) and the differentiation of peripheral Tregs (pTregs) (42, 43). Tregs are associated with poor prognosis in HCC, breast cancer, colorectal cancer and other solid tumors (44, 45). In addition to suppressing natural killer (NK) cells and CD8+ cytotoxic T cells, they contribute to tumor progression by promoting vascular invasion, tumor cell migration and proliferation, and inhibiting tumor cell apoptosis (46, 47). In a DEN-induced HCC mouse model, blocking the TGFβ receptor on Tregs reduced their infiltration and hindered HCC progression (41). In another study, Treg depletion led to anti-tumor immune response in 38% of human HCC cases (48).

Tregs inhibit T cells by IL-6 and IL-17 secretions (49). Previous studies in human HCC have reported both negative and positive correlations between Tregs and CD8+ T cells (37). Although, we did not observe strong correlations between Tregs and CD8+ T cells in the IM or tumor regions, strong negative correlation was observed between Tregs in the IM and CD8+ T cells in the tumor. This can be explained by previous studies showing that increased Tregs reduce CD8+ T cells, preventing their infiltration into tumors via IL-10, IL-35 and TGFβ (42, 50). The pronounced accumulation of Tregs in the IM region, alongside an inverse correlation with intratumoral CD8+ T cells, supports the hypothesis that the IM region is a key site of immune suppression. This mechanistic insight into regional immune cell distribution suggests that targeting Tregs within the IM might enhance antitumor immunity and improve therapeutic outcomes.

In HCC liver, complex interactions were observed between the T lymphocyte and macrophage populations. In the IM region, KCs and Inf mphs positively correlated with CD8+ T cells, suggesting a cooperative anti-tumor response, while in tumor region, Tregs negatively correlated with KCs, suppressing this response. Overall, the interplay between T cells and macrophages highlights a dynamic immune landscape, where CD8+ T cells and macrophages work together to promote anti-tumor activity, while Tregs suppress these responses, facilitating tumor progression.

In both rat and human models, it has been demonstrated that the enzymatic activity and functionality varies depending on liver zonation, with cytochrome P450 predominantly concentrated in the CV region (51). Moreover, it has been previously studied that CCl_4_ treatment induces centrilobular hepatocyte cell death and extracellular matrix deposition leading to liver fibrosis (34). As a model confirmation, we compared the immune infiltration between the CV and periportal regions of the study group. And observed an increased Inf mph, CD3+ T cells, CD4+ T cells, and Tregs in the CV region in the HCC-NTT, indicating the relevance of advanced fibrosis and immune dysregulation (**Supplementary Figure S2**).

Previous research in humans has characterized HCC into distinct immune subclasses such as “active” (effector T cell enrichment) and “exhausted immune” (T cell exhaustion and immunosuppressive macrophages) (22). Other classifications have categorized HCC into immune “high,” “mid,” and “low,” or as “immunocompetent” (normal T cell infiltration), “immunosuppressive” (high CD45, regulatory T and B cells and immunosuppressive macrophages) and “immunodeficient” (reduced infiltration of lymphocytes) based on the infiltration and activity of the immune cells (11, 52, 53). Our model can be considered as the immunosuppressive subclass of HCC.

## Conclusion

In conclusion, our DEN-CCl_4_ mouse model of CLD and HCC progression uniquely revealed the dynamic shifts in immune cell populations. The elevated levels of Tregs, and Inf mphs, combined with a lack of increase of intratumoral CD8+ T cells in the HCC livers, closely resembled the immunosuppressive type of human HCC. The increased presence of immune cells in the IM region, highlights its role as a critical interface where tumor cells interact with the surrounding parenchyma, while the rise in the NTT indicates significant immune dysregulation in advanced CLD potentially promoting tumor growth. Our study highlights the critical role of the IM region in HCC, where Tregs are abundant and inversely correlated with CD8^+^ T cells, suggesting that targeted depletion of Tregs and enrichment/reprogramming of Inf mphs toward a pro-inflammatory phenotype could restore effective anti-tumor immunity. The DEN-CCl_4_ model offers a promising preclinical platform to evaluate these spatially targeted immunotherapies, which hold potential for clinical application in overcoming immune suppression within the IM and improving outcomes for HCC patients.

### Limitations and future prospects

In this study, we focused on a chemically induced HCC model (DEN-CCl_4_), which captures key features of the immunosuppressive subtype. However, investigating additional models-such as those mimicking viral or metabolic liver diseases-would provide deeper insights into the heterogeneity and diverse etiologies of human HCC. Spatial profiling of key immune cells, such as myeloid and lymphoid populations, provides important insights into how immune suppression evolves during CLD.

Integrating single-cell RNA sequencing with spatial transcriptomics offers a promising avenue to deepen our understanding of immune heterogeneity in HCC, particularly by characterizing distinct NK, B, and Treg cell subsets within the tumor microenvironment. Specifically, profiling NK cells can reveal their suppressive states under TGF-β influence from Tregs, while detailed B cell subset analysis, including Bregs versus effector B cells, could clarify their contrasting roles in tumor progression. Furthermore, dissecting Treg heterogeneity and their spatial distribution across tumor, IM, and NTT regions will help in deciphering their contributions to immunosuppression. Incorporating functional experiments-such as depleting or reprogramming immune cells-along with transcriptomic and proteomic analyses, will be crucial for understanding how tumor cells interact with immune cells, particularly within the IM region. These approaches will help validate the immunosuppressive mechanisms and elucidate the molecular crosstalk between tumor cells, immune cells, and the inflammatory microenvironment. Together, these future directions will advance our understanding of liver cancer immunobiology and support the development of more effective, personalized immunotherapies.

## Data Availability

The raw data supporting the conclusions of this article will be made available by the authors, without undue reservation.
