# Supplementary material for: Progression to fibrosis and hepatocellular carcinoma in DEN CCl4 liver mice, is associated with macrophage and striking regulatory T cells infiltration
Source: Front Immunol. 2025 Jul 8;16:1601215. doi: 10.3389/fimmu.2025.1601215 (PMC12279789; doi:10.3389/fimmu.2025.1601215)
Supplement: Supplementary file 1 [file DataSheet1.docx]

**Stained cell detection by Qupath**

Regions of interest (ROIs) in control, fibrosis, and HCC liver tissues were annotated, excluding outer capsular regions and large blood vessels for uniform tissue analysis.

In HCC samples, tumor and invasive margin (IM) regions were manually outlined. Tumor borders were identified based on alpha-smooth muscle actin (α-SMA) immunostaining and differences in cell size between tumor and non-tumor (NTT) parenchyma. IM was delineated at 300-500 μm from the tumor border, based on tumor size (tumor area 10^7^- 20^7^μm^2^ – IM expansion radius of 300μm, tumor area >40^7^μm^2^ – IM expansion radius of 500μm) (Fig S1). Portal tracts (PT) were differentiated from the central vein (CV) by the CK19 immunostaining of cholangiocytes, with PT and CV regions manually outlined and expanded by 100 μm to study immune cell infiltration. Cell segmentation was performed using Stardist tool with trained cell classifiers applied for each marker. Immunostained cell density was calculated as the number of detected cells/mm² tissue area for each ROI, enabling quantification of immune cell infiltration and distribution across tissue ([Berben](https://onlinelibrary.wiley.com/authored-by/Berben/Lieze) et al., 2020, [Galon](https://pathsocjournals.onlinelibrary.wiley.com/authored-by/Galon/Jérôme) et al., 2013, Kirilovskyet al., 2016).


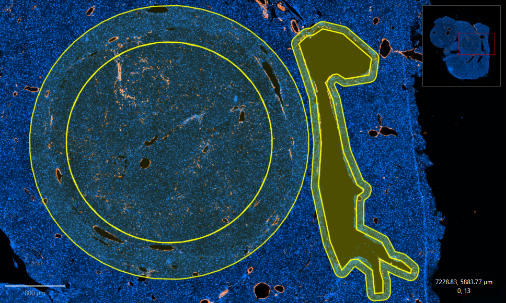


**Tumour**

**IM**

**Vessel**

**NTT**

**A**


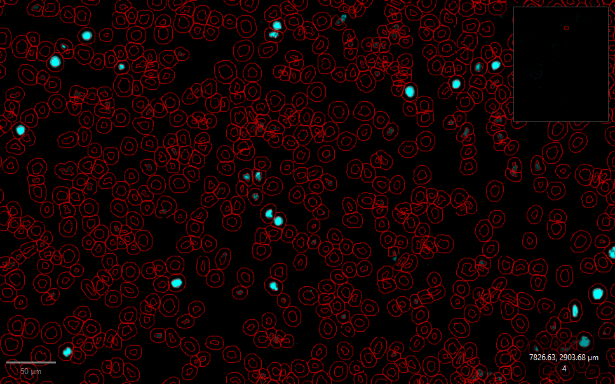


**B**


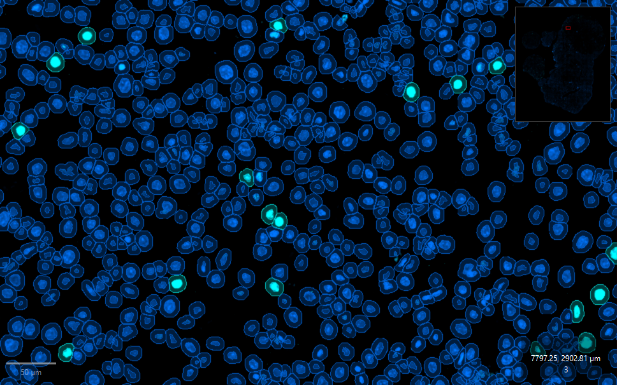


**C**

Fig S1: (A) Within HCC liver, ROI was trained to identify the total tissue. Thereafter, the vessels and tumor regions were manually outlined. IM region includes the border between the tumor and remaining non-tumorigenic tissue and this was determined by expanding the annotation radius up to 500μm. (B) cell detection of Ki67 (Cyan) and other cells of the liver tumor. stained (cyan- Ki67) and unstained (blue- Hoechst) cell types were correctly identified by training the classifier accordingly.

**REFERENCES**

1. Berben, L., Wildiers, H., Marcelis, L., Antoranz, A., Bosisio, F., Hatse, S., & Floris, G. (2020). Computerized scoring protocol for identification and quantification of different immune cell populations in breast tumor regions by the use of QuPath software. *Histopathology*, *77*(1), 79-91.
2. Galon, J., Mlecnik, B., Bindea, G., Angell, H. K., Berger, A., Lagorce, C., ... & Pagès, F. (2014). Towards the introduction of the ‘Immunoscore’in the classification of malignant tumors. *The Journal of pathology*, *232*(2), 199-209.
3. Kirilovsky, A., Marliot, F., El Sissy, C., Haicheur, N., Galon, J., & Pagès, F. (2016). Rational bases for the use of the Immunoscore in routine clinical settings as a prognostic and predictive biomarker in cancer patients. *International immunology*, *28*(8), 373-382.
