# Supplementary material for: Progression to fibrosis and hepatocellular carcinoma in DEN CCl4 liver mice, is associated with macrophage and striking regulatory T cells infiltration
Source: Front Immunol. 2025 Jul 8;16:1601215. doi: 10.3389/fimmu.2025.1601215 (PMC12279789; doi:10.3389/fimmu.2025.1601215)
Supplement: Supplementary file 2 [file DataSheet2.docx]

**Immune cell infiltration increases at the central vein region, which is the site of inflammation in fibrosis and remaining tumor tissue of HCC liver**

Given that CV region is the site of inflammation for CCl_4_ induced hepatic injury model (S. Hammad et al., 2017), we further characterized the localization of different hepatic immune cell populations in this zone. Absolute numbers of leukocytes, myeloid cells, granulocytes, CD3^+^ T cells, CD8^+^ cytotoxic T cells, Tregs, resident KCs, and Inf mphs were assessed in CV and PT regions across healthy, fibrosis, and HCC-NTT livers. In HCC-NTT regions, CD45^+^ leukocytes and CD11b^+^ myeloid cells were significantly enriched near the CV compared to PT regions (Fig S2.A, S2.B). KC numbers remained constant across regions and groups, but Inf mph showed a steady increase in both CV and PT regions, with more prominent abundance in the HCC-NTT. In healthy liver, Inf mphs represented less than 20% of the total hepatic macrophage populations, but this ratio was increased 3 to 4-fold as the liver disease progresses from fibrosis to advanced CLD and HCC (Fig S2.C, S2.D, S2.E). Granulocytes showed no significant changes in distribution across the groups, remaining sparse throughout. An overall increase in the density of CD3^+^ T cells at the CV regions was observed in fibrosis and NTT-HCC livers as compared to healthy liver. Although, no significant change in the cytotoxic CD8^+^ T cell numbers were observed both near the CV or PT regions, CD4^+^ T cells and Tregs infiltration increased significantly around the CV of advanced CLD regions in the HCC model as compared to the other two groups (Fig S2.F, S2.G).


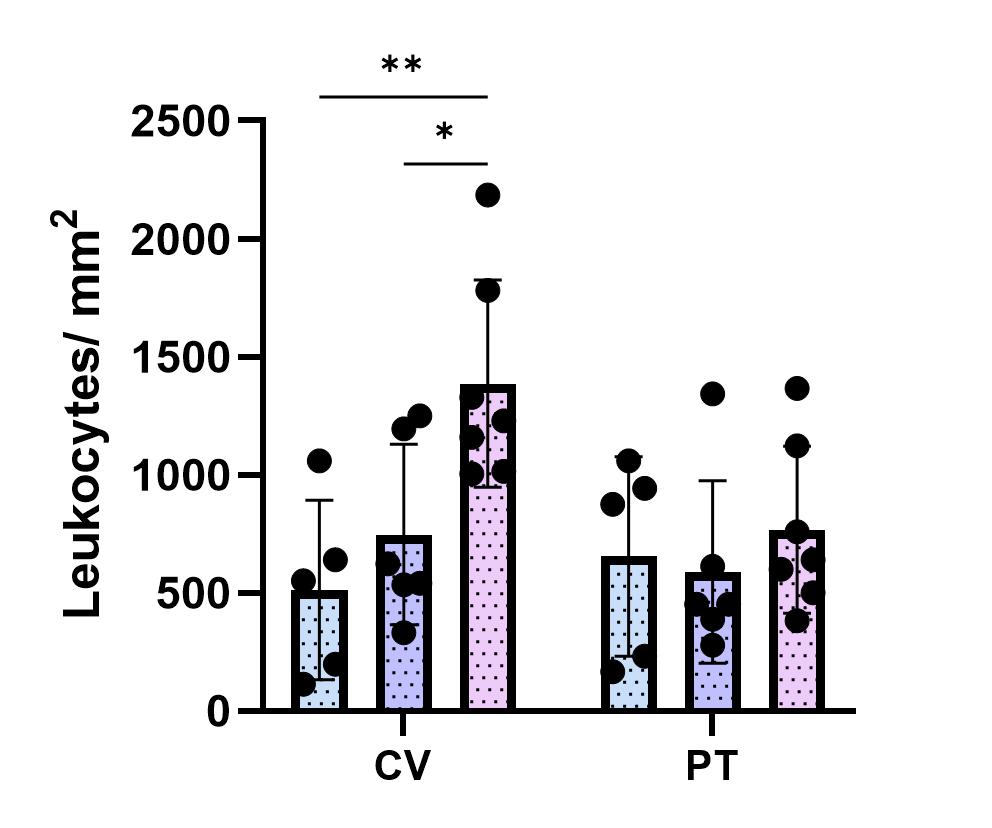

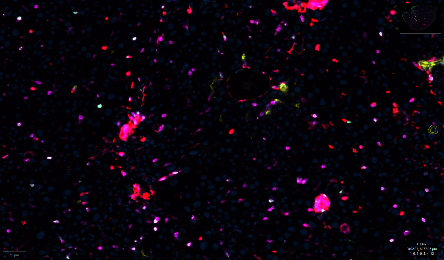

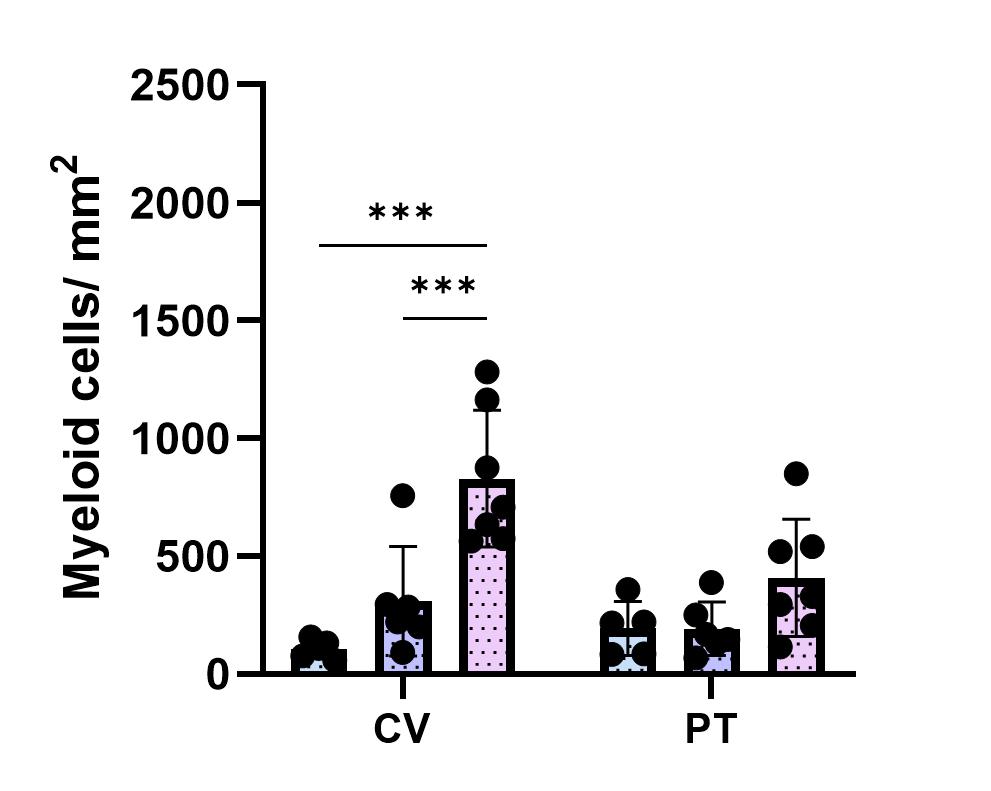

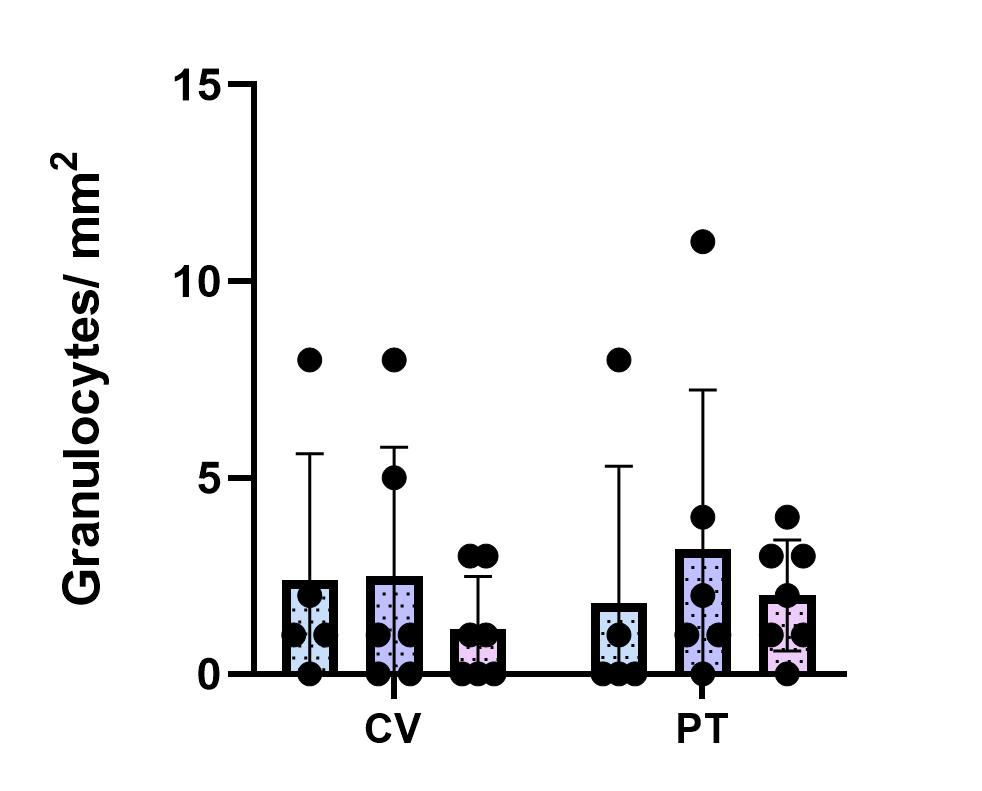


**PT**

**CV**

DAPI CK19 αSMA CD45 CD3 CD11b

**A**

**B**


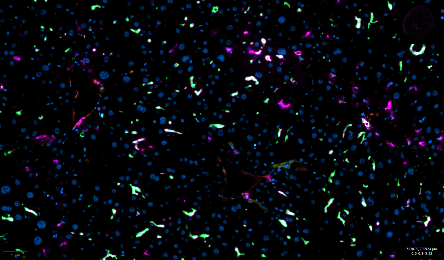


**PT**

**CV**

DAPI CK19 αSMA Iba1 CLEC4F

**C**

**D**

**E**


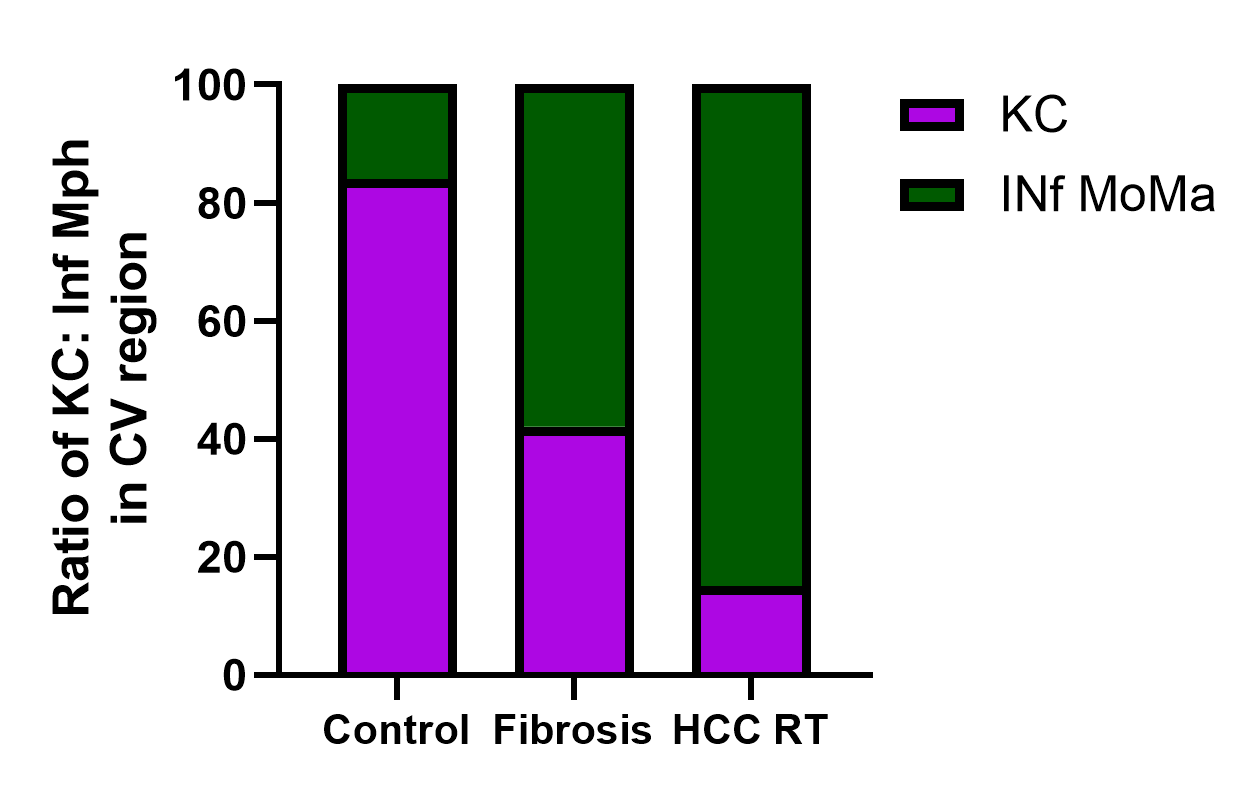

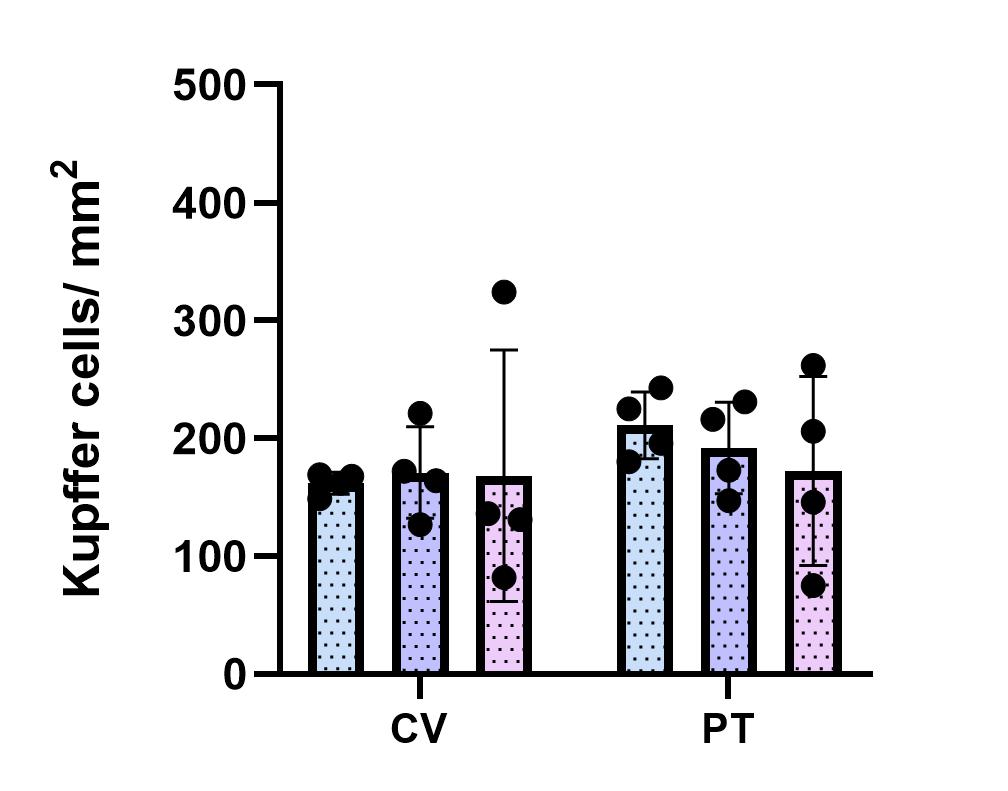

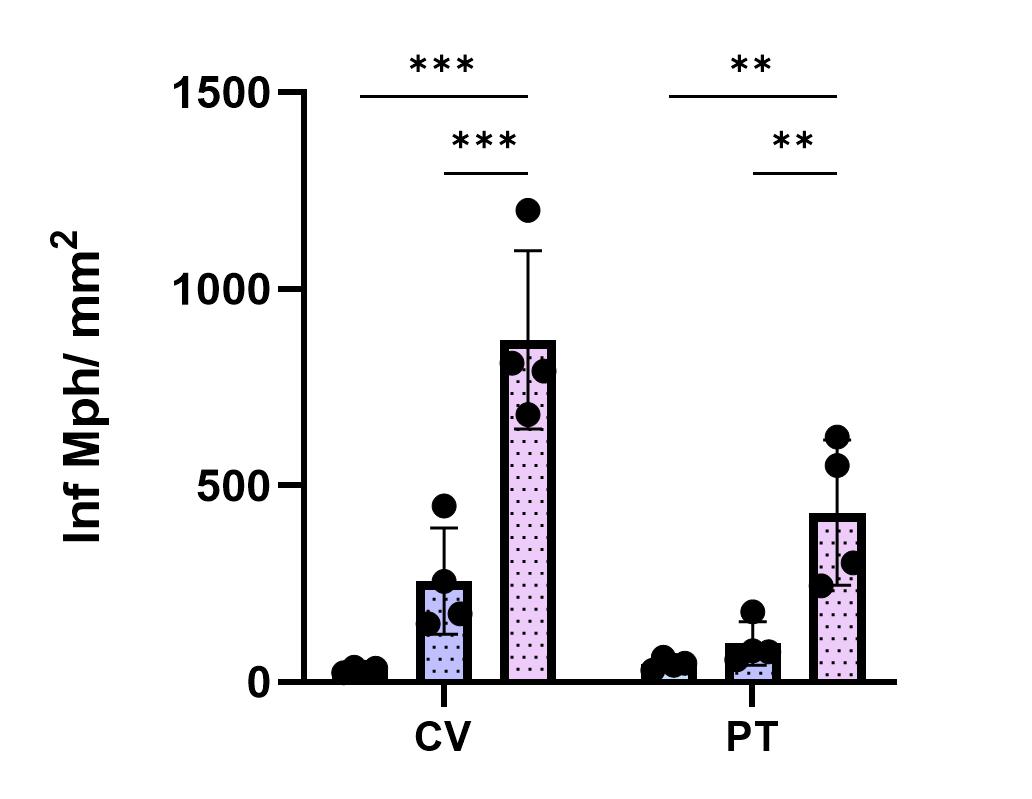

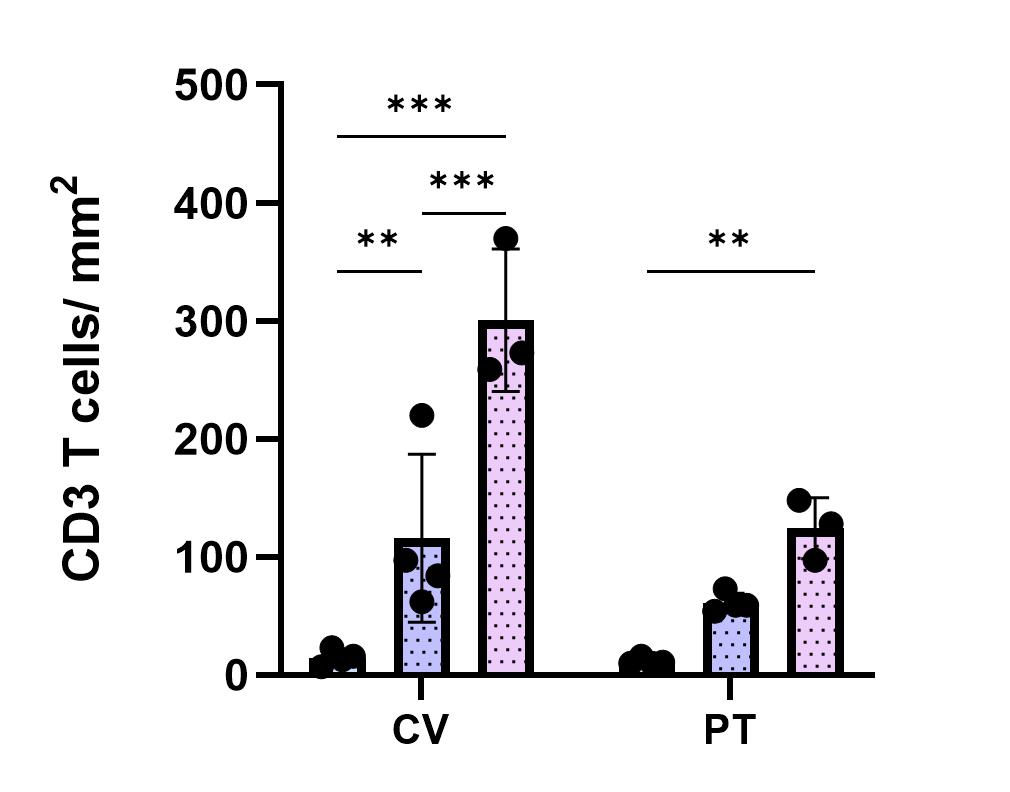

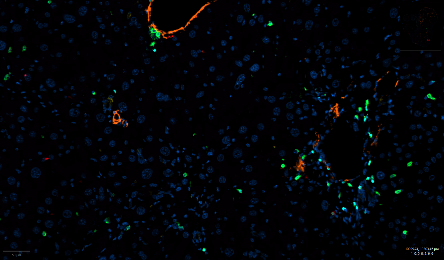


**PT**

**CV**

DAPI CK19 αSMA CD3 CD8 FoxP3

**F**

**G**


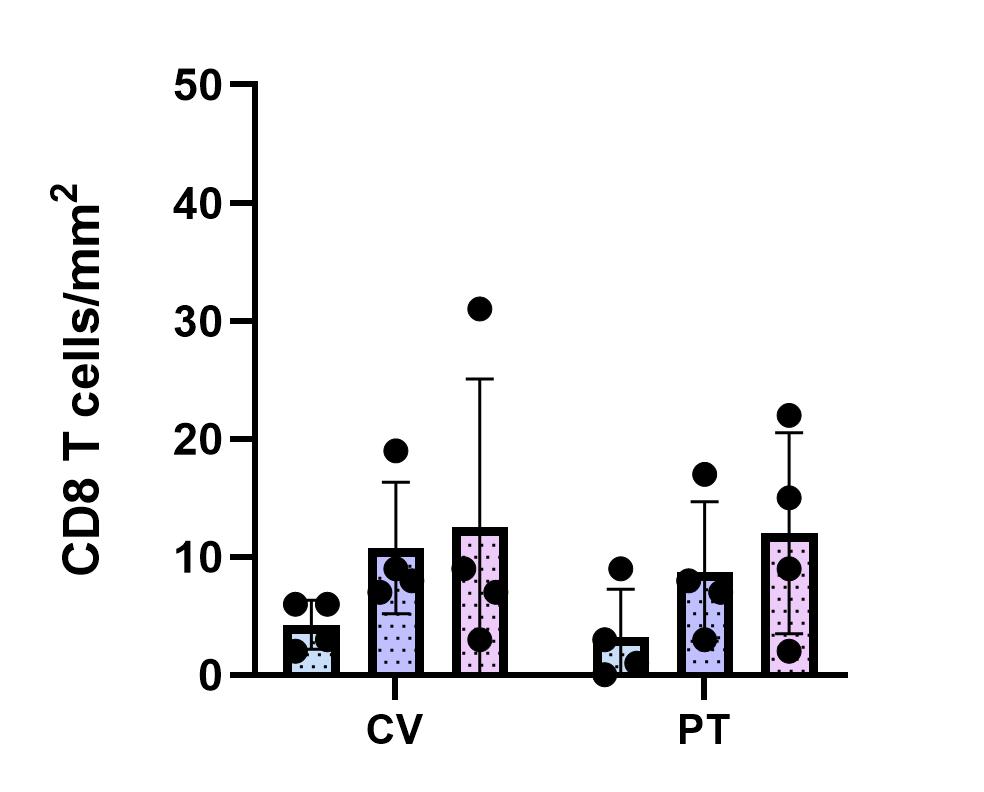

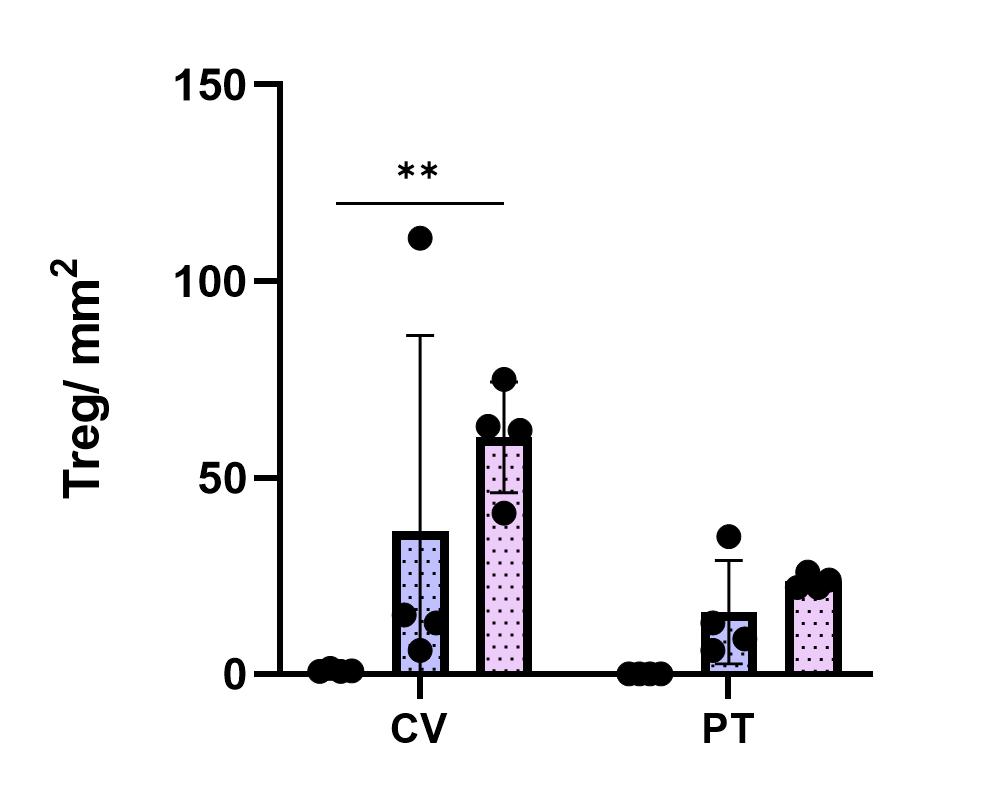

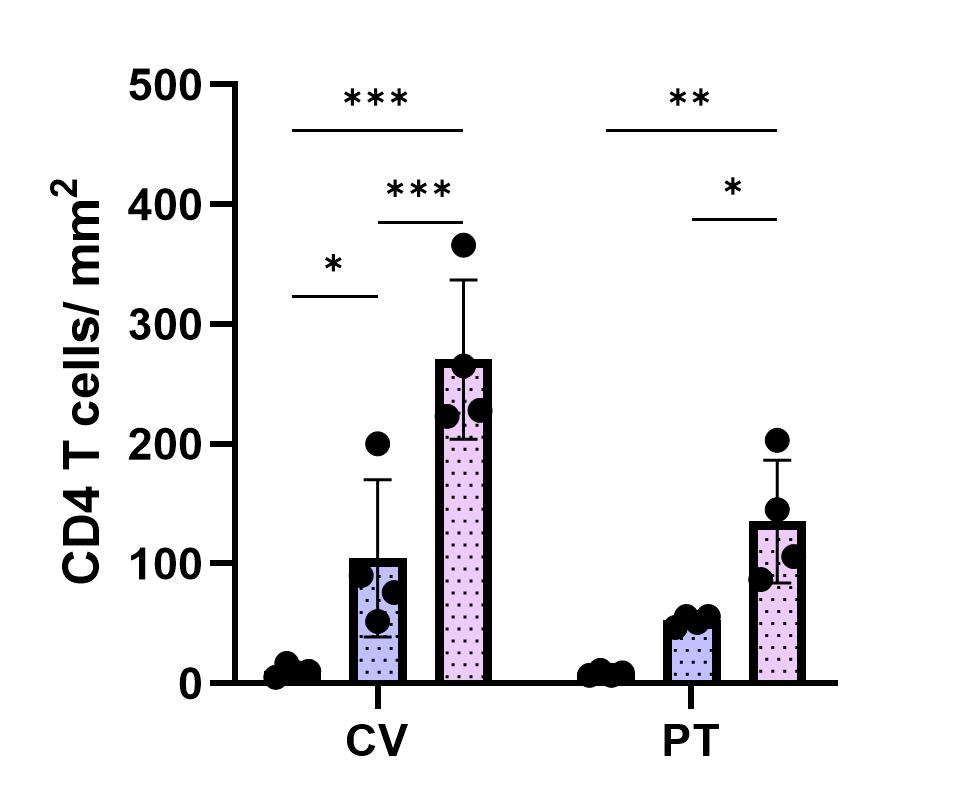

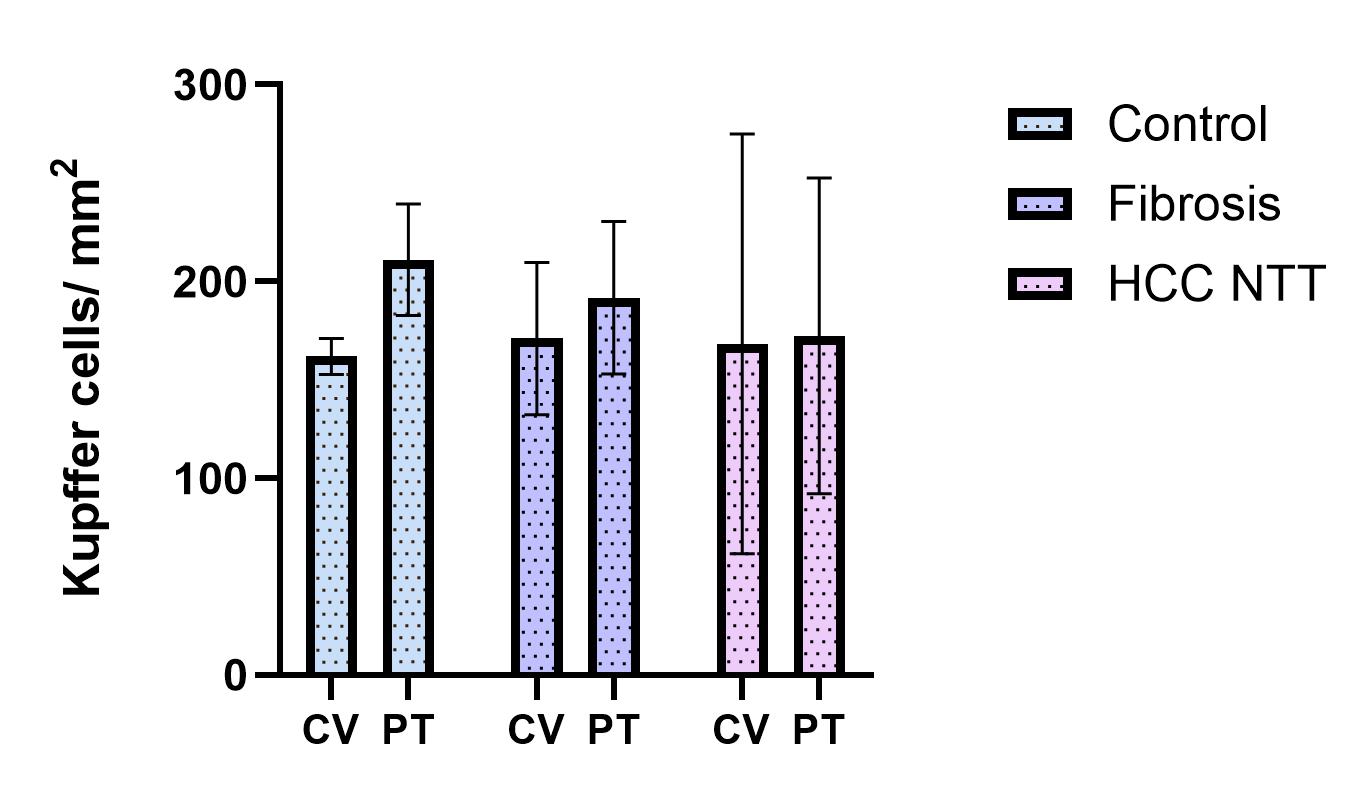

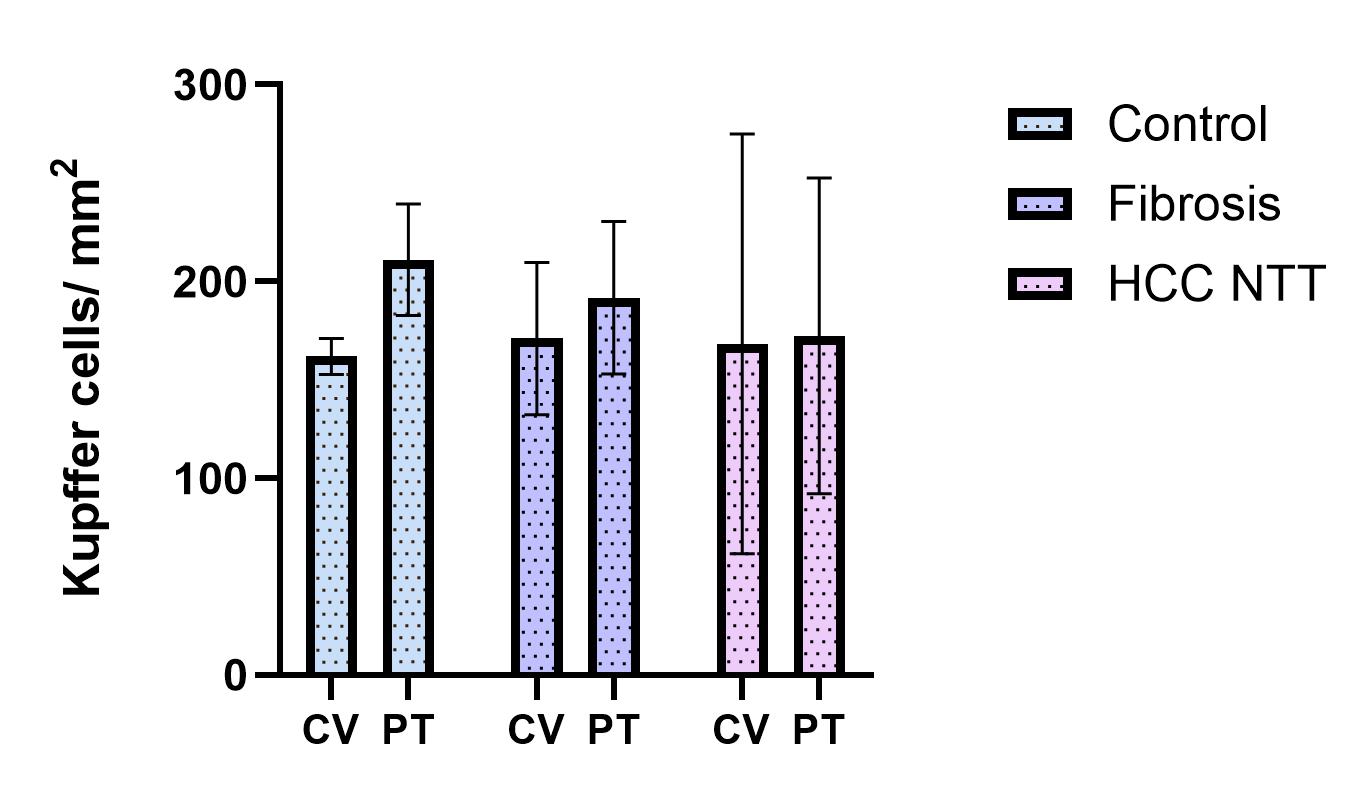

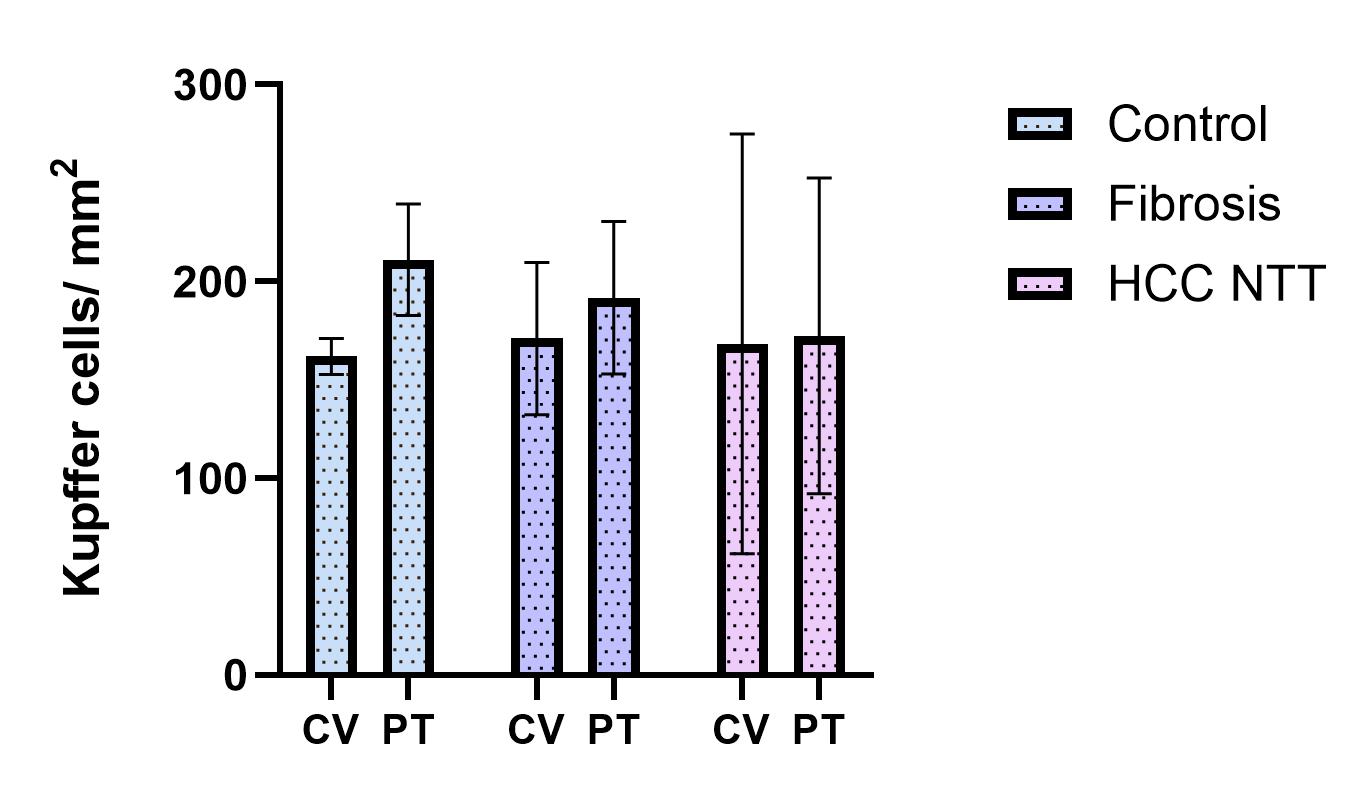


**Figure S2. Increased infiltration of different subsets of immune cells near CV, the site of inflammation in CCl4 treated liver damage.**

**A**, Representative multiplex IF image showing the spatial distribution of leukocytes, myeloid cells and granulocytes around the CV and PT regions. **B**, Number of leukocytes, myeloid cells and granulocytes per mm^2^ tissue area around CV and PT in control (n=5), fibrosis (n=6) and HCC-NTT (n=7). **C**, Representative multiplex IF image showing the spatial distribution of CD3 T, CD8 T cells and Tregs around the CV and PT regions. **D**, Number of CD3 T cells, CD8 T cells and Tregs per mm^2^ tissue area around CV and PT in control (n=4), fibrosis (n=4) and HCC-NTT (n=4). **E,** Representative multiplex IFC image showing the spatial distribution of KCs and Inf Mphs around the CV and PT regions. **F**, Number of KCs (Iba1^+^ Clec4F^+^) and Inf Mphs (Iba1^+^ Clec4F^-^) per mm^2^ tissue area around CV and PT in control (n=4), fibrosis (n=4) and HCC-NTT (n=4). **G**, Ratio of KCs and Inf Mphs present around the CV, the site of inflammation.

**REFERENCE**

1. Hammad, S., Braeuning, A., Meyer, C., Mohamed, F. E. Z. A., Hengstler, J. G., & Dooley, S. (2017). A frequent misinterpretation in current research on liver fibrosis: the vessel in the center of CCl4-induced pseudolobules is a portal vein. *Archives of Toxicology*, *91*(11), 3689-3692.
