# Supplementary material for: Progression to fibrosis and hepatocellular carcinoma in DEN CCl4 liver mice, is associated with macrophage and striking regulatory T cells infiltration
Source: Front Immunol. 2025 Jul 8;16:1601215. doi: 10.3389/fimmu.2025.1601215 (PMC12279789; doi:10.3389/fimmu.2025.1601215)
Supplement: Supplementary file 3 [file Table1.docx]

**Table S1- List of antigens used to identify the different cell types for multiplex IF**

| **Antigen** | **Cell type detected** |
| --- | --- |
| HNF4^+^ | Hepatocytes |
| HNF4^+^, Ki67^+^ | Proliferating hepatocytes |
| CD45^+^ | Leukocytes |
| CD45^+^ Ki67^+^ | Proliferating leukocytes |
| CD11b^+^ | Myeloid cells |
| Ly6G^+^ | Granulocytes |
| IBA1^+^ | Hepatic macrophages |
| IBA1^+^, CLEC4F^+^ | Kupffer cells |
| IBA1^+^, CLEC4F^+^, Ki67^+^ | Proliferating Kupffer cells |
| IBA1^+^, CLEC4F^+^ | Infiltrating macrophages |
| IBA1^+^, CLEC4F^-^, Ki67^+^ | Proliferating Infiltrating macrophages |
| CD3^+^ | Leukocytes |
| CD8^+^ | CD8^+^ T cells |
| CD3^+^, CD8^-^ | CD4^+^ T cells |
| FoxP3^+^ | Tregs |
