# Supplementary material for: Progression to fibrosis and hepatocellular carcinoma in DEN CCl4 liver mice, is associated with macrophage and striking regulatory T cells infiltration
Source: Front Immunol. 2025 Jul 8;16:1601215. doi: 10.3389/fimmu.2025.1601215 (PMC12279789; doi:10.3389/fimmu.2025.1601215)
Supplement: Supplementary file 4 [file Table2.docx]

**Table S2- Multiplex IF panel, antibodies and fluorescence**

| **Antigen** | **Primary antibody** | | | | **Secondary antibody** | **Tyramide** | | |
| --- | --- | --- | --- | --- | --- | --- | --- | --- |
|  | **Dilution** | **Company** | **Reference** | **HIER method** | **Polymer HRP** | **Company** | **Reference** | **Fluorochrome** |
| **PANEL 1: Ki67** |  |  |  |  |  |  |  |  |
| **Ki67**  (Clone SP6) | 1/250 | Abcam | Ab16667 | Citrate  (pH6) | Anti-rabbit | Biotium | 92171 | CF488 |
| **HNF4**  (Clone 4C19) | 1/2500 | Sigma | ZRB1457-25UL | Tris-EDTA  (pH9) | Anti-rabbit | ThermoFisher | B40957 | AF594 |
| **CK19**  (Clone TROMA-III) | 1/20 | DSHB | TROMA-III | Citrate  (pH6) | Rabbit anti-rat  +  Anti-rabbit | Biotium | 96021 | CF555 |
| **CD45**  (Clone D3F8Q) | 1/150 | Cell Signaling | 70257 | Citrate  (pH6) | Anti-rabbit | Biotium | 96090 | CF754 |
| **αSMA**  (Polyclonal) | 1/200 | Abcam | Ab5694 | Citrate  (pH6) | Anti-rabbit | Biotium | 96022 | CF647 |
| **PANEL 2: Leukocytes** |  |  |  |  |  |  |  |  |
| **CK19**  (Clone TROMA-III) | 1/20 | DSHB | TROMA-III | Citrate  (pH6) | Rabbit anti-rat  +  Anti-rabbit | Biotium | 96021 | CF555 |
| **Ly6G**  (Clone E6Z1T) | 1/900 | Cell Signaling | 87048 | Tris-EDTA  (pH9) | Anti-rabbit | Biotium | 96053 | CF430 |
| **CD3**  (Clone D4V8L) | 1/50 | Cell Signaling | 99940 | Citrate  (pH6) | Anti-rabbit | Biotium | 92171 | CF488 |
| **CD45**  (Clone D3F8Q) | 1/100 | Cell Signaling | 70257 | Citrate  (pH6) | Anti-rabbit | Biotium | 96090 | CF754 |
| **CD11b**  (Clone E4K8C) | 1/200 | Cell Signaling | 93169 | Citrate  (pH6) | Anti-rabbit | ThermoFisher | B40957 | AF594 |
| **αSMA**  (Polyclonal) | 1/200 | Abcam | Ab5694 | Citrate  (pH6) | Anti-rabbit | Biotium | 96022 | CF647 |
| **PANEL 3: Macrophages** |  |  |  |  |  |  |  |  |
| **Cleaved caspase-3** (Clone 269518) | 1/1000 | R&D Systems | MAB835 | Tris-EDTA  (pH9) | Anti-rabbit | ThermoFisher | B40957 | AF594 |
| **Clec4F**  (Clone 370901) | 1/30 | R&D Systems | MAB2784 | Citrate  (pH6) | Rabbit anti-rat  +  Anti-rabbit | Biotium | 92171 | CF488 |
| **Ki67**  (Clone SP6) | 1/500 | Abcam | Ab16667 | Citrate  (pH6) | Anti-rabbit | Biotium | 96053 | CF430 |
| **IBA1**  (Clone E4O4W) | 1/500 | Cell Signaling | 17198 | Citrate  (pH6) | Anti-rabbit | Biotium | 96090 | CF754 |
| **αSMA**  (Polyclonal) | 1/200 | Abcam | Ab5694 | Citrate  (pH6) | Anti-rabbit | Biotium | 96022 | CF647 |
| **CK19**  (Clone TROMA-III) | 1/20 | DSHB | TROMA-III | Citrate  (pH6) | Rabbit anti-rat  +  Anti-rabbit | Biotium | 96021 | CF555 |
| **PANEL 4: Lymphocytes** |  |  |  |  |  |  |  |  |
| **Foxp3**  **(Clone D6O8R)** | 1/100 | Cell Signaling | 12653 | Citrate  (pH6) | Anti-rabbit | Biotium | 96053 | CF430 |
| **CD3**  **(Clone D4V8L)** | 1/50 | Cell Signaling | 99940 | Citrate  (pH6) | Anti-rabbit | Biotium | 92171 | CF488 |
| **αSMA**  **(Polyclonal)** | 1/200 | Abcam | Ab5694 | Citrate  (pH6) | Anti-rabbit | Biotium | 96022 | CF647 |
| **CD8**  **(Clone D4W2Z)** | 1/100 | Cell Signaling | 98941 | Citrate  (pH6) | Anti-rabbit | ThermoFisher | B40957 | AF594 |
| **CD137**  **(Clone E2J5H)** | 1/50 | Cell Signaling | 18798 | Tris-EDTA  (pH9) | Anti-rabbit | Biotium | 96090 | CF754 |
| **CK19**  **(Clone TROMA-III)** | 1/20 | DSHB | TROMA-III | Citrate  (pH6) | Rabbit anti-rat  +  Anti-rabbit | Biotium | 96021 | CF555 |
