# Supplementary material for: Progression to fibrosis and hepatocellular carcinoma in DEN CCl4 liver mice, is associated with macrophage and striking regulatory T cells infiltration
Source: Front Immunol. 2025 Jul 8;16:1601215. doi: 10.3389/fimmu.2025.1601215 (PMC12279789; doi:10.3389/fimmu.2025.1601215)
Supplement: Supplementary file 5 [file Table3.docx]

**Table S3- Flow cytometry antibodies and fluorescence**

| **Reagent** | **Manufacturer** | **Identifier** |
| --- | --- | --- |
| CD45 Antibody, anti-mouse, APC-Vio® 770, REAfinity™ | Miltenyi | 130-110-662  REA737 |
| CD11b Antibody, anti-mouse, Vio® Bright FITC, REAfinity™ | Miltenyi | 130-113-243  REA592 |
| F4/80 Antibody, anti-mouse, APC, REAfinity™ | Miltenyi | 130-123-913  REA126 |
| Ly6C Antibody, anti-mouse, VioGreen, REAfinity™ | Miltenyi | 130-111-784  REA796 |
| Brilliant Violet 650™ anti-mouse Ly-6G Antibody | BioLegend | 127641  1A8 |
| Brilliant Violet 711™ anti-mouse CD3 Antibody | BioLegend | 100241  17A2 |
| CD4 Antibody, anti-mouse, Vio® Bright FITC, REAfinity™ | Miltenyi | 130-118-692  REA604 |
| CD8a Antibody, anti-mouse, PerCP-Vio® 700, REAfinity™ | Miltenyi | 130-128-616  REA601 |
| FoxP3 Antibody, anti-mouse, PE, REAfinity™ | Miltenyi | 130-111-678  REA788 |
| FcR Blocking Reagent, mouse | Miltenyi | 130-092-575 |
| FoxP3 Staining Buffer Set | Miltenyi | 130-093-142 |
| DAPI | Cell Signaling | 4083 |
