# Supplementary material for: Progression to fibrosis and hepatocellular carcinoma in DEN CCl4 liver mice, is associated with macrophage and striking regulatory T cells infiltration
Source: Front Immunol. 2025 Jul 8;16:1601215. doi: 10.3389/fimmu.2025.1601215 (PMC12279789; doi:10.3389/fimmu.2025.1601215)
Supplement: Supplementary file 6 [file Table4.docx]

**Table S4- Spearman’s correlation analysis in tumor, IM, and NTT regions of HCC liver**

| **Positive** |  |  |
| --- | --- | --- |
| **Correlations** | **r value** | **p value** |
| Tumor % Ki67+ cells v/s IM Ki67+ hepatocytes | 0,642857 | 0,020757 |
| Tumor % Ki67+ cells v/s Tumor Ki67+ leukocytes | 0,587912 | 0,038063 |
| Tumor % Ki67+ cells v/s Tumor Ki67+ KC | 0,594971 | 0,035397 |
| IM % Ki67+ cells v/s IM Ki67+ hepatocytes | 0,814577 | 0,000697 |
| IM % Ki67+ cells v/s IM Ki67+ leukocytes | 0,725055 | 0,004718 |
| IM KC v/s IM Ki67+ cells | 0,777584 | 0,004112 |
| IM KC v/s IM Ki67+ hepatocytes | 0,727273 | 0,009631 |
| Tumor Ki67+ Inf mph v/s T Ki67+ hepatocytes | 0,678626 | 0,012958 |
| IM Ki67+ leukocytes v/s IM Ki67+ Hepatocytes | 0,85873 | 0,00019 |
| NTT myeloid cells v/s NTT Ki67+ hepatocytes | 0,82143 | 0,03413 |
| Tumor myeloid cells v/s Tumor leukocytes | 0,857909 | 9,16E-05 |
| IM myeloid cells v/s IM leukocytes | 0,585259 | 0,030434 |
| IM granulocytes v/s IM leukocytes | 0,563349 | 0,038176 |
| IM KC v/s IM leukocytes | 0,605955 | 0,040274 |
| NTT myeloid cells v/s NTT leukocytes | 0,821429 | 0,034127 |
| IM KC v/s IM Ki67+ leukocytes | 0,70753 | 0,01246 |
| Tumor KC v/s Tumor Mph | 0,60989 | 0,030207 |
| IM Inf mph v/s IM Mph | 0,76224 | 0,00553 |
| Tumor Ki67+ Inf mph v/s Tumor Ki67+ KC | 0,717497 | 0,007856 |
| IM Ki67+ Inf mph v/s IM Ki67+ KC | 0,893171 | 0,000213 |
| IM CD8+ T cells v/s IM Ki67+ KC | 0,662567 | 0,021796 |
| Tumor CD8+ T cells v/s Tumor Ki67+ Inf mph | 0,601942 | 0,032375 |
| IM CD8+ T cells v/s IM Ki67+ Inf mph | 0,729829 | 0,009101 |
| Tumor CD4+ T cells v/s Tumor CD3+ T cells | 0,637363 | 0,022138 |
| Tumor Treg v/s Tumor CD3+ T cells | 0,63173 | 0,023292 |
| IM CD4+ T cells v/s IM CD3+ T cells | 0,97521 | 2,35E-07 |
| IM Treg v/s IM CD3+ T cells | 0,729656 | 0,006012 |
| IM CD4+ T cells v/s Tumor CD4+ T cells | 0,60989 | 0,030207 |
| IM Treg v/s IM CD4+ T cells | 0,662999 | 0,015903 |

| **Negative** |  |  |
| --- | --- | --- |
| **Correlations** | **r value** | **p value** |
| IM Inf mph v/s IM Ki67+ hepatocytes | -0,62937 | 0,032296 |
| Tumor Treg v/s Tumor KC | -0,56276 | 0,04815 |
| IM Treg v/s Tumor CD8+ T cells | -0,63071 | 0,023489 |
| IM CD8+ t cells v/s Tumor CD4+ T cells | -0,65285 | 0,01802 |
| IM Inf mph v/s IM KC | -0,76223 | 0,005533 |
| IM CD4+ T cells v/s Tumor CD8+ T cells | -0,60222 | 0,032708 |
